# Arthropod transcriptional activator protein-1 (AP-1) aids tick-rickettsial pathogen survival in the cold

**DOI:** 10.1038/s41598-018-29654-6

**Published:** 2018-07-30

**Authors:** Supreet Khanal, Vikas Taank, John F. Anderson, Hameeda Sultana, Girish Neelakanta

**Affiliations:** 1 Department of Biological Sciences, Old Dominion University, Norfolk, VA USA; 2 Department of Entomology, Connecticut Agricultural Experiment Station, New Haven, CT USA; 3 Center for Molecular Medicine, Old Dominion University, Norfolk, VA USA

## Abstract

*Ixodes scapularis* ticks transmit several pathogens to humans including rickettsial bacterium, *Anaplasma phagocytophilum*. Here, we report that *A*. *phagocytophilum* uses tick transcriptional activator protein-1 (AP-1) as a molecular switch in the regulation of arthropod antifreeze gene, *iafgp*. RNAi-mediated silencing of *ap-1* expression significantly affected *iafgp* gene expression and *A*. *phagocytophilum* burden in ticks upon acquisition from the murine host. Gel shift assays provide evidence that both the bacterium and AP-1 influences *iafgp* promoter and expression. The luciferase assays revealed that a region of approximately 700 bp upstream of the antifreeze gene is sufficient for AP-1 binding to promote *iafgp* gene expression. Furthermore, survival assays revealed that AP-1-deficient ticks were more susceptible to cold in comparison to the mock controls. In addition, this study also indicates arthropod AP-1 as a global regulator for some of the tick genes critical for *A*. *phagocytophilum* survival in the vector. In summary, our study defines a novel mode of arthropod signaling for the survival of both rickettsial pathogen and its medically important vector in the cold.

## Introduction

Human anaplasmosis is one of the most common arthropod-borne diseases in the United States. This disease is caused by the rickettsial bacteria *Anaplasma phagocytophilum*^1–3^. *A*. *phagocytophilum* is transmitted to humans by a bite from an infected blacklegged tick, *Ixodes scapularis*. In the northeastern part of the United States, these ticks spend most of their life off the hosts in a natural microenvironment that also includes survival in extreme cold and sub-zero temperatures^1,4,5^. The life cycle of *I*. *scapularis* involves four stages: eggs, larvae, nymphs and adults. Larval ticks hatch from eggs in late summer and fall, take a blood meal upon feeding on small vertebrate hosts, and molt into nymphs^1,4,5^. Nymphs overwinter and take a blood meal in late spring or early summer of the following year and molt into large size adult male or female ticks. Adults overwinter and female ticks take a blood meal and mate with the male ticks to lay eggs in the following year. Most humans acquire infections from nymphal tick bites^2,6,7^.

Upon ingestion, *A*. *phagocytophilum* enters the gut and then colonizes the salivary glands with persistent infection^1,7^. *A*. *phagocytophilum* is not transmitted transovarially. These bacteria are maintained transstadially including overwintering stages^6–11^. The arthropod molecular signaling that *A*. *phagocytophilum* modulates to survive in its vector host during overwintering stages is not understood.

In our previous study, we reported the identification of a novel antifreeze molecule from ticks^12^ and designated it as IAFGP (*I*. *scapularis* antifreeze glycoprotein). We also noted that *A*. *phagocytophilum* specifically induces *iafgp* expression and increases the ability of these ticks to survive better in cold^12^. RNAi studies showed the importance of IAFGP for the survival of *I*. *scapularis* ticks in a cold environment^12^. In addition, expression of IAFGP rendered cold tolerance in mice and flies^13,14^. Our previous study also reported that *A*. *phagocytophilum* changes tick cytoskeleton by altering the ratio of monomeric/filamentous (G/F) actin to transcriptionally regulate expression of *salp16*, a gene crucial for the bacterial survival^15^. Recently, we showed that *A*. *phagocytophilum* modulates expression of tick organic anion transporters and tryptophan pathways for its survival in ticks^16^. Several other recent studies have extensively focused on understanding modulation of signaling pathways during *A*. *phagocytophilum*-tick interactions^17–22^. However, studies in delineating molecular gene regulation of this important tick gene (*iafgp*) upon *A*. *phagocytophilum* infection and survival in ticks during cold are not yet described.

The genome of *I*. *scapularis* encodes several transcription factors^23^. Activator protein-1 (AP-1) family of transcription factors are important modulators of several cellular processes including differentiation, proliferation, immune regulation and apoptosis^24–30^. In humans, AP-1 family comprises of homo- and hetero-dimer members of protein families that include Jun (c-Jun, JunB and JunD), Fos (c-Fos, FosB, Fra-1 and Fra-2), ATF (ATF2, ATF3/LRF1, B-ATF, JDP1 and JDP2) and MAF (c-Maf, MafB, MafA, MafG/K and Nrl)^25–29^. These proteins are characterized by the presence of a conserved dimeric basic leucine zipper DNA-binding domain^25,26^. AP-1 complex binds specific promoters of several genes either to activate or repress them^25,26,28^. In addition, AP-1 family of proteins are important to maintain the basal expression of several genes^31^.

AP-1 is a highly conserved transcription factor across wide range of organisms^32–34^. Recent studies have reported roles for vertebrate AP-1 in host-pathogen interactions including a role in *Chlamydia pneumoniae* persistence in human epithelial cells^30,34,35^. The role of AP-1 in tick biology and/or in the interactions of ticks with pathogens remains to be elucidated. In this study, we show that *A*. *phagocytophilum* uses arthropod AP-1 molecule as a molecular switch to regulate tick antifreeze gene that is important for both vector and pathogen survival in the cold.

## Results

### Genomic organization of *iafgp* gene

**Figure 1:**
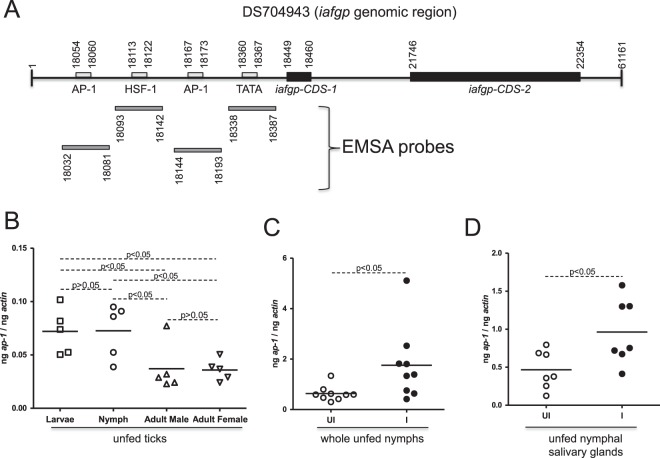
Activator protein *ap-1* mRNA is upregulated in *A*. *phagocytophilum*-infected unfed nymphs. (**A**) Genomic region in *I*. *scapularis* 1108462552178 genomic scaffold derived from whole genome shotgun sequence (GenBank accession number DS704943) is shown. Rectangular boxes on the top of the bold line indicates AP-1, HSF-1 binding sites or TATA region. Location and position of sequences used to generate EMSA probes are indicated as grey bars below the bold line. The *iafgp* gene sequences are distributed in two putative exons (*iafgp*-CDS-1 and *iafgp*-CDS-2) shown as closed black boxes on the bold line. All position numbers correspond to GenBank acc. no. DS704943. The schematic representation of genomic region is not to the scale. QRT-PCR assay results showing levels of *ap-1* transcripts in naïve unfed larvae, nymphs, adult male and adult female ticks (**B**) in uninfected (UI) and *A*. *phagocytophilum*-infected (I) whole nymphs (**C**) or in salivary glands (**D**) isolated from unfed nymphs is shown. Data for larvae in panel B was generated from pooled tick samples (5–7 ticks/pool). In panel B, each triangle, circle or inverted triangle represents one tick sample. In panel C and D, each open circle indicates samples from uninfected ticks (UI) and closed circle indicates samples from *A*. *phagocytophilum*-infected ticks. The levels of *ap-1* transcripts were normalized to the levels of tick beta-actin transcripts. Statistical analysis was performed using Student’s t test and P value less than 0.05 was considered significant in all panels (B–D).

Analysis of the genomic region in *I*. *scapularis* 1108462552178 genomic scaffold, derived from whole genome shotgun sequence (GenBank acc. no. DS704943) revealed that the coding sequences (CDS) of *iafgp* gene is distributed in two putative (CDS-1 and CDS-2) exons (Fig. 1A). The total length of DS704943 genomic scaffold is 61161 bp. Using bioinformatics tools (as mentioned in the methods), a strong TATA region (TATATAAT, 18360–18367 bp) in the putative *iafgp* promoter region was identified. Analysis for the presence of putative transcription factor binding sites using TFBIND^36^ revealed more than 100 different predicted sites for various transcription factors. These include putative activator protein-1 (AP-1) binding sites (TGACGCT 18054–18060 bp, TGTCGCA 18167–18173 bp) and heat shock factor-1 (HSF-1) binding site (TGTGCCTTCC, 18113–18122). The consensus sequence for the TATA box on the eukaryotic promoter is TATA(A/T)A(A/T)(A/G)^37^. The consensus sequences for AP-1 binding region is TGAC/GTCA^38^. HSF proteins bind specifically to heat shock sequence elements (HSE) whose consensus sequence consists of three 5-mer sites of AGAAN or a degenerate version of this sequence^39^. As AP-1 site is close to the putative *iafgp* TATA box, its role in vector-pathogen interactions and as a global regulator^30,34,35^, further studies were carried out on tick AP-1.

### Developmental and pathogen-mediated regulation of *ap-1* in ticks

We first assessed whether unfed uninfected ticks express *ap-1* transcripts at different developmental stages. Ticks encode *ap-1* gene in its genome (GenBank acc. no. XM_002404527). The Quantitative RT-PCR (QRT-PCR) analysis revealed higher levels of *ap-1* mRNA in larvae and nymphs (P < 0.05) when compared to both adult males and females (Fig. 1B). The differences in the *ap-1* transcripts between larvae and nymphs were not significant (P > 0.05) (Fig. 1B). Comparison of *ap-1* mRNA levels between male and females also revealed no significant (P > 0.05) differences (Fig. 1B). These results indicate differential regulation of *ap-1* mRNA at various developmental stages of ticks. Furthermore, analysis in whole unfed nymphs (Fig. 1C) or in salivary glands (Fig. 1D) or guts (Supplementary Fig. 1) isolated from unfed nymphs (Fig. 1D) revealed higher levels (P < 0.05) of *ap-1* transcripts upon *A*. *phagocytophilum* infection of ticks when compared to the uninfected controls.

### Comparative analysis of *I*. *scapularis* AP-1 with other AP-1-like proteins from hard ticks

**Figure 2:**
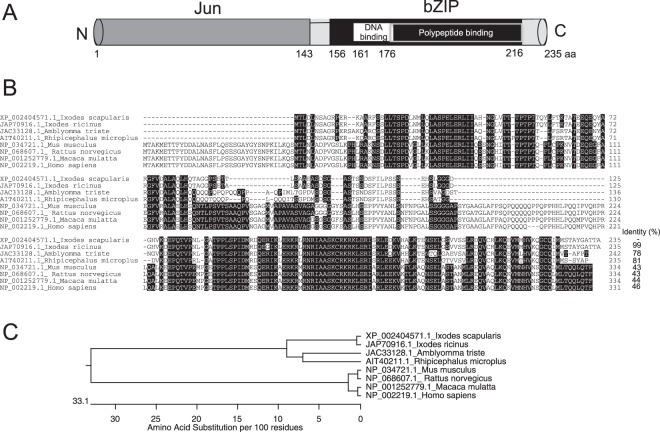
Alignment and phylogenetic analysis of *Ixodes scapularis* AP-1 with other orthologs. (**A**) Schematic representation of AP-1 protein structure is shown. Domain analysis of *I*. *scapularis* AP-1 primary amino acid sequence showing presence of Jun domain (1–143 aa) at N-terminus and bZIP domain at C-terminus (156–216 aa). In addition, DNA binding domain (161–176 aa) and polypeptide-binding domain (175–216 aa) were evident at the C-terminus region. (**B**) *I*. *scapularis* AP-1 amino acid sequence alignment (with other orthologs) using ClustalW program in DNASTAR (Lasergene Genomics Suite) is shown. Residues that match are shaded in black color. GenBank accession numbers for *I*. *ricinus*, *Amblyomma triste*, *Rhipicephalus microplus*, *Mus musculus*, *Rattus norvegicus*, *Macaca mulatta and Homo sapiens* sequences are shown on the left side. Total length and percent identities of the amino acid sequences compared with *I*. *scapularis* AP-1 are provided at one end of each sequence. (**C**) Phylogenetic tree was generated in DNASTAR by ClustalW slow/accurate alignment method using Gonnet as default value for protein weight matrix. Scale at the bottom denotes amino acid substitutions per 100 amino acid residues.

We used annotated sequence (GenBank acc. no. XP_002404571) of tick *ap-1* mRNA to analyze AP-1 protein domain(s) structure. The domain structure revealed that the putative AP-1 protein contained a Jun domain (1–143 amino acids) at the N-terminal end and basic leucine zipper (bZip) domain (156–216 aa) at the C-terminal end (Fig. 2A). Within the bZip domain, DNA-binding domain (161–176 aa) and polypeptide-binding domain (175–216 aa) were evident (Fig. 2A). BLAST search followed by ClustalW alignment (using DNASTAR) revealed presence of AP-1 like orthologs in other ticks (Fig. 2B). These include *I*. *ricinus* (GenBank acc. no. JAP70916), *Amblyomma triste* (GenBank acc. no. JAC33128) and *Rhipicephalus microplus* (GenBank acc. no. AIT40211). High degree of conservation across the entire amino acid sequence was evident in *I*. *scapularis* AP-1 in comparison to AP-1 from other ticks. Where, *I*. *scapularis* AP-1 shared an identity of 99% with *I*. *ricinus*, 78% with *A*. *triste* and 81% with *R*. *microplus* AP-1 orthologs (Fig. 2B). Furthermore, we noted that *I*. *scapularis* AP-1 shared an identity of 43% with *Mus musculus* (house mouse, GenBank acc. no. NP_034721) and *Rattus norvegicus* (rat, GenBank acc. no. NP_068607) AP-1 and 44% with *Macaca mulatta* (Rhesus monkey) AP-1 (GenBank acc. no. NP_001252779) and 46% with *Homo sapiens* (human) AP-1 (GenBank acc. no. NP_002219) orthologs (Fig. 2B). ClustalW alignment also showed absence of some of the sequences at the N-terminal end of tick AP-1 molecules in comparison to the mammalian orthologs (Fig. 2B). Phylogenetic tree derived from the ClustalW alignment revealed that *I*. *scapularis* AP-1 falls within the same clade along with other tick AP-1 orthologs (Fig. 2C). Where as, AP-1 from mice, rats, monkey and humans forms different clade (Fig. 2C).

### *A*. *phagocytophilum* influences *iafgp* promoter through *I*. *scapularis* AP-1

**Figure 3:**
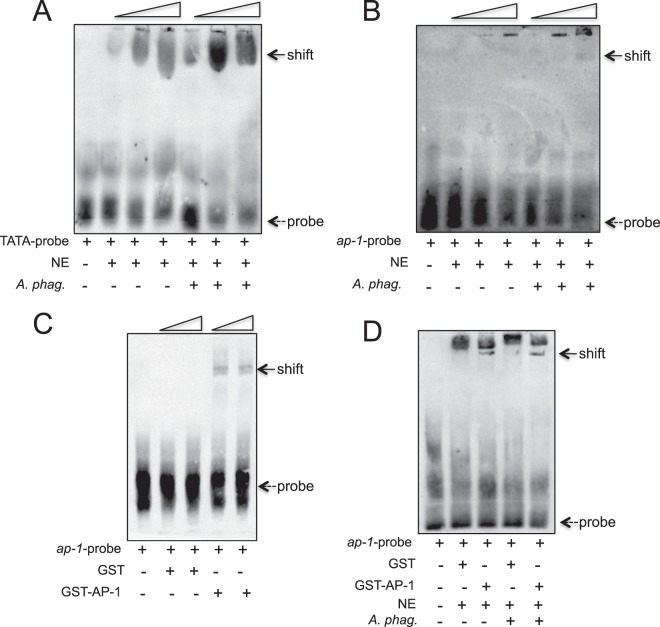
*A*. *phagocytophilum* and AP-1 influence *iafgp* promoter. (**A**) Gel shift assay results showing enhanced band intensity in the shift of *iafgp* TATA-probe upon incubation with nuclear proteins isolated from *A*. *phagocytophilum-*infected nymphs in comparison to the shift observed upon incubation with nuclear proteins isolated from uninfected nymphs. The biotinylated *iafgp* TATA-binding region promoter probe (DS704943, 18338–18387 bp) and nuclear proteins from uninfected or *A*. *phagocytophilum*–infected nymphs were used in EMSAs. Wedges represent increase in the amount of nuclear proteins (1, 3, 5 µg). (**B**) Gel shift assays with biotinylated *iafgp* promoter probe containing AP-1 binding site (DS704943, 18144–18193 bp) and nuclear proteins (1, 2, 3 µg in increasing order as indicated by wedges) from uninfected or *A*. *phagocytophilum*–infected nymphs is shown. (**C**) Gel shift assays with biotinylated *iafgp* promoter probe containing AP-1 binding site (DS704943, 18144–18193 bp) and recombinant GST or rGST-AP-1 protein (1, 1.5 µg, in increasing order as indicated by wedges) is shown. (**D**) Gel shift assays with biotinylated *iafgp* promoter probe containing AP-1 binding site (DS704943, 18144–18193 bp), rGST alone or rGST-AP-1 protein (1.5 µg) and nuclear proteins (3 µg) prepared from uninfected or *A*. *phagocytophilum*–infected nymphs is shown. Dotted arrow indicates free probe and solid arrow indicates the shift. NE indicates nuclear extracts and + or − indicates presence or absence, respectively.

Previously, we reported that *A*. *phagocytophilum* induces *iafgp* expression in ticks^12^. However, the molecular mechanism of this gene regulation was not elucidated. To provide an evidence for the *iafgp* gene regulation, we performed gel shift or electrophoretic mobility shift assays (EMSAs). Total nuclear protein lysates from uninfected or *A*. *phagocytophilum*-infected unfed ticks was prepared and tested its binding on biotin-labeled DNA probe (position corresponding to 18338–18387 in GenBank acc. no. DS704943) containing putative *iafgp* TATA promoter region (Fig. 1A). The shift in the biotin-labeled DNA probe indicates binding of nuclear protein(s) to the probe. EMSAs indicated an approximately two to three fold increase in the shift of *iafgp* TATA probe upon incubation with total nuclear proteins isolated from *A*. *phagocytophilum*-infected ticks when compared to the incubation with nuclear proteins isolated from uninfected ticks (Fig. 3A and Supplementary Fig. 2A,B). Incubation of biotin-labeled *iafgp* promoter region DNA probe (position corresponding to 18144–18193 in GenBank acc. no. DS704943) containing putative AP-1 binding site (Fig. 1A) revealed two to three fold increased shift upon incubation with total nuclear proteins isolated from *A*. *phagocytophilum*-infected ticks when compared to incubation with nuclear proteins isolated from uninfected ticks (Fig. 3B and Supplementary Fig. 2C,D). However, biotin-labeled *iafgp* promoter region DNA probe (position corresponding to 18032–18081 in GenBank acc. no. DS704943) containing another putative AP-1 binding site (Fig. 1A) did not show any shift upon incubation with nuclear lysates prepared from either groups (Supplementary Fig. 3A,B). In addition, biotin-labeled *iafgp* promoter region DNA probe (position corresponding to 18093–18142 in GenBank acc. no. DS704943) containing putative heat shock factor-1 (HSF-1) binding site (Fig. 1A) also did not reveal a shift upon incubation with lysates prepared from either groups (Supplementary Fig. 3C,D). These results indicate that *A*. *phagocytophilum*-mediated influence on *iafgp* promoter is dependent on TATA region (position corresponding to 18360–18367 in GenBank acc. no. DS704943) and a specific AP-1 binding region (position corresponding to 18167–18173 in GenBank acc. no. DS704943).

Furthermore, to elucidate whether *I*. *scapularis* AP-1 directly binds the AP-1 binding site (position corresponding to 18167–18173 in GenBank acc. no. DS704943) in the *iafgp* promoter region, the probe indicated in Fig. 1A and used in Fig. 3B was incubated with recombinant *I*. *scapularis* AP-1 (rGST-AP-1) conjugated with glutathione S-transferase (Supplementary Fig. 4A,B). EMSAs revealed a clear shift in the AP-1 probe upon incubation with rGST-AP-1 (Fig. 3C and Supplementary Fig. 5A,B). As expected, no shift was observed upon incubation of AP-1 probe with rGST alone (Fig. 3C and Supplementary Fig. 5A,B). Incubation of AP-1 probe with rGST-AP-1 and nuclear proteins isolated from *A*. *phagocytophilum*-infected ticks revealed a increased and a clear shift in comparison to the faint shift observed upon incubation of AP-1 probe with rGST and nuclear proteins isolated form *A*. *phagocytophilum*-infected ticks (Fig. 3D and Supplementary Fig. 5C,D). The observation of faint shift with rGST is due to the nuclear proteins (possibly higher levels of endogenous AP-1) present in the lysates prepared from *A*. *phagocytophilum*-infected ticks and not due to GST. The observation of no shift in the AP-1 probe upon incubation with rGST in the presence of nuclear proteins isolated from uninfected ticks (Fig. 3D and Supplementary Fig. 5C,D) further supports this data. These results clearly suggest that nuclear lysates prepared from *A*. *phagocytophilum*-infected ticks contain higher levels of AP-1.

**Figure 4:**
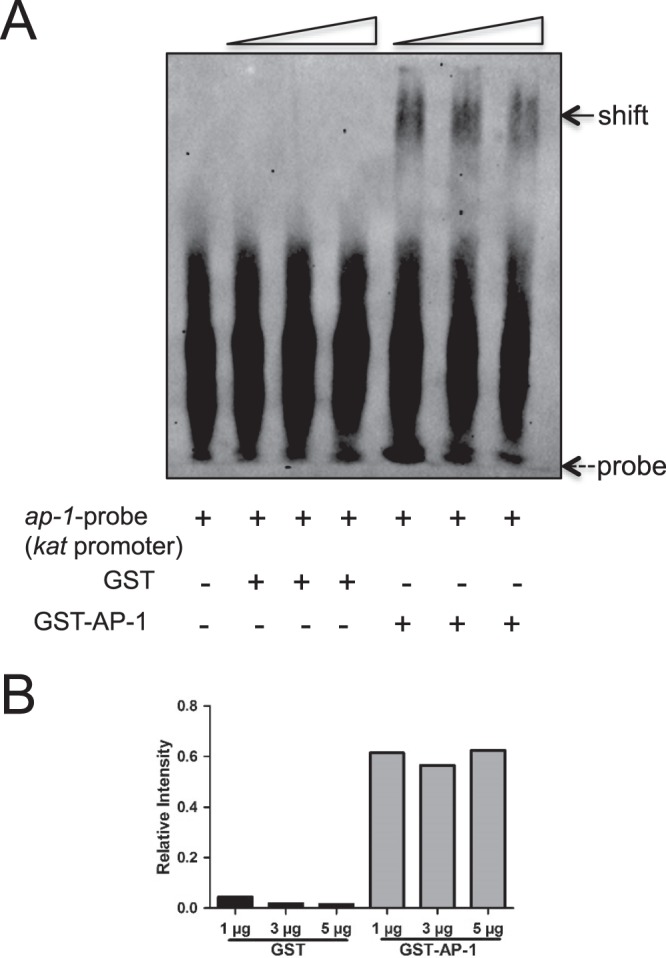
AP-1 binds *kat gene* promoter. (**A**) EMSAs performed with the biotinylated AP-1 region probe (DS929842, 124879–124830 bp) from *kat* putative promoter containing AP-1 binding site (DS929842, 124858–124852 bp) and rGST alone or rGST-AP-1 protein (1, 3, 5 µg; wedges indicates increasing amounts of nuclear extracts). Dotted arrow indicates free probe and solid arrow indicates band shift. + indicates presence and − indicates absence. (**B**) Densitometry analysis for the gel image in (**A**) is shown. Relative intensities of gel shifts were calculated to the control probe intensity in each gel image.

As AP-1 is a global regulatory molecule, we tested whether *A*. *phagocytophilum* influence AP-1 to regulate some of the tick genes critical for its survival in ticks. In our previous study, we reported that *A*. *phagocytophilum* modulates expression of organic anion transporting polypeptide (OATP) and kynurenine aminotransferase (KAT) pathway for its survival in ticks^16^. Based on bioinformatics analysis, a strong AP-1 binding site (TGAATCA, GenBank acc. no. DS929842, 124858–124852 bp) was noted in the putative *kat* promoter region. EMSAs performed with biotinylated AP-1 probe (GenBank acc. no. DS929842, 124879–124830 bp) from *kat* promoter and with rGST or rGST-AP-1 revealed a strong gel shift in the presence of AP-1 (Fig. 4A,B and Supplementary Fig. 6). As expected no shift was observed when the same probe was incubated with rGST alone (Fig. 4A,B and Supplementary Fig. 6). Taken together, these results clearly confirm that the identified putative site on the *iafgp* promoter (position corresponding to 18167–18173 in GenBank acc. no. DS704943) or *kat* promoter (GenBank acc. no. DS929842, 124858-124852 bp) is indeed an AP-1-binding region and suggests the important role of AP-1 as a global transcriptional activator in the regulation of essential tick genes critical for *A*. *phagocytophilum* survival in the vector host.

### Region of approximately 700 bp upstream of the *iafgp* coding sequence is sufficient *for A. phagocytophilum*-mediated influence on tick antifreeze gene expression

**Figure 5:**
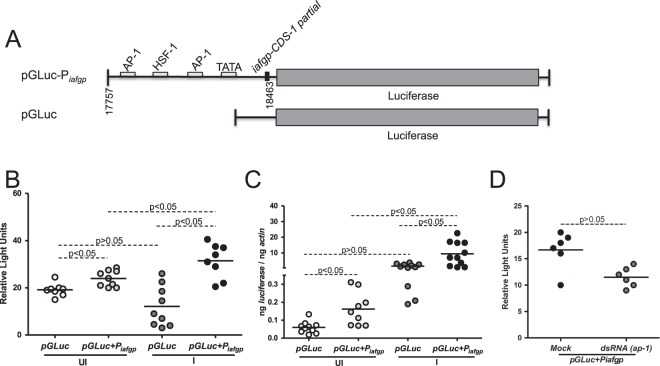
Approximately 700 bp DNA sequence upstream of tick antifreeze gene is sufficient to drive *iafgp* gene expression. (**A**) Schematic representation of the *iafgp* promoter construct used to analyze promoter activity in tick cells. The schematics is not drawn to scale. ~700 bp DNA sequence (position corresponding to 17757–18463 in GenBank acc. no. DS704943) upstream of *iafgp* gene containing all tested binding sites (AP-1, HSF-1, TATA-region) was cloned into promoterless pGLuc (pGLuc-P_*iafgp*_) vector. The empty pGLuc vector was used as control. These constructs were transfected into tick cells and luciferase assay was performed. (**B**) Luciferase activity measurements determined from culture supernatants from uninnfected (UI) or *A*. *phagocytophilum*-infected (I) ISE6 cells transfected with pGLuc-P_*iafgp*_ or pGLuc constructs is shown. (**C**) Levels of luciferase transcripts in uninfected (UI) or *A*. *phagocytophilum*-infected (I) ISE6 cells transfected with pGLuc-P_*iafgp*_ or pGLuc constructs is shown. The levels of luciferase transcripts were normalized to the levels of beta-actin transcripts. Open or dark grey circles represents uninfected (UI) or *A*. *phagocytophilum*-infected (I) cells transfected with pGluc alone, respectively. Light grey or black circles represents uninfected (UI) or *A*. *phagocytophilum*-infected (I) cells transfected with pGLuc-P_*iafgp*_, respectively. (**D**) Luciferase activity measurements determined from culture supernatants from *A*. *phagocytophilum*-infected ISE6 cells treated with mock-dsRNA or *ap-1-*dsRNA and transfected with pGLuc-P_*iafgp*_ constructs is shown. In panels B and D, each circle represents luciferase activity measurement from supernatants collected from one culture well. In panel C, each circle indicates sample generated from cells from one culture well. Statistical analysis was performed using Student’s t test and P value less than 0.05 was considered significant in panels B–D.

As EMSAs revealed a strong TATA and AP-1 binding site regions upstream of the *iafgp* CDS-1, we amplified and sequenced ~700 bp upstream region from *I*. *scapularis* genomic DNA (Supplementary Fig. 7A,B). This 700 bp fragment (position corresponding to 17757–18463 in GenBank acc. no. DS704943) containing putative *iafgp* promoter region was cloned in pGLuc promoterless vector (Fig. 5A) and processed for luciferase assays as mentioned in the methods. The uninfected or *A*. *phagocytophilum*-infected tick cells showed no morphological differences upon transfection of pGLuc vector carrying *iafgp* promoter (pGLuc + P_*iafgp*_) or empty pGLuc vector (Supplementary Fig. 8). Enhanced (P < 0.05) luciferase activity was observed in both uninfected and *A*. *phagocytophilum*-infected tick cells transfected with pGLuc + P_*iafgp*_ in comparison to respective control cells transfected with an empty vector (Fig. 5B). Luciferase activity was approximately two fold increased (P < 0.05) in *A*. *phagocytophilum*-infected tick cells upon transfection with pGLuc + P_*iafgp*_ in comparison to the uninfected tick cells transfected with pGLuc + P_*iafgp*_ (Fig. 5B). In addition, luciferase mRNA was detected in tick cells transfected with either of the constructs (Supplementary Fig. 9A). QRT-PCR analysis measuring luciferase mRNA in each group further supported the results from luciferase promoter assays (Fig. 5C). Furthermore, significantly (P < 0.05) decreased luciferase activity was observed upon transfection of pGLuc + P_*iafgp*_ construct *in A*. *p*hagocytophilum-infected *ap-1*-silenced tick cells in comparison to the mock-dsRNA treated tick cells (Fig. 5D). Collectively, these results indicate that region of ~700 bp upstream of the *iafgp* coding sequence is sufficient for *A*. *phagocytophilum*-mediated AP-1-dependent regulation of tick antifreeze gene.

### RNAi of *ap-1* affects *iafgp* expression and *A*. *phagocytophilum* burden in nymphs during acquisition

**Figure 6:**
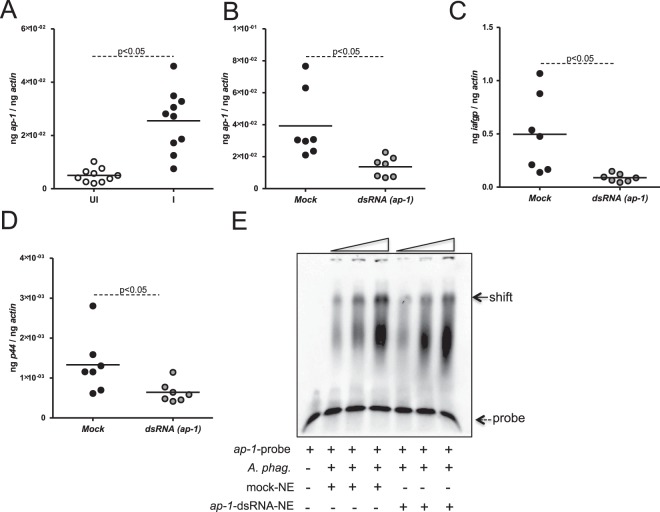
Knockdown of *ap-1* affects bacterial acquisition and *iafgp* gene expression in ticks. (**A**) QRT-PCR results showing levels of *ap-1* transcripts in nymphs fed on uninfected or *A*. *phagocytophilum*-infected mice. The levels of *ap-1* (**B**) or *iafgp* (**C**) transcripts in mock or *ap-1*-deficient ticks upon bacterial acquisition from murine host are shown. (**D**) Bacterial burden in 48 h post-repleted mock or *ap*-1-deficient nymphs fed on *A*. *phagocytophilum*-infected mice is shown. (**E**) Gel shift assays with biotinylated *iafgp* promoter probe containing AP-1 binding site (DS704943, 18144–18193 bp) and nuclear proteins (2, 4, 6 µg in increasing order as indicated by wedges on the top of the image) prepared from *A*. *phagocytophilum*-infected mock or *ap-1*-deficient ticks is shown. Dotted arrow indicates free probe and solid arrow indicates shift. Each circle in panel A–D indicates individual tick. Statistical analysis was performed using Student’s t test and P value less than 0.05 was considered significant in panels A–D. NE indicates nuclear extracts and + or − indicates presence or absence, respectively.

We next analyzed *ap-1* mRNA levels in uninfected nymphal ticks upon feeding on either naïve or *A*. *phagocytophilum*-infected murine host. We found significantly increased (P < 0.05) *ap-1* mRNA levels in ticks that were engorged on *A*. *phagocytophilum*-infected mice when compared to the levels noted in ticks that fed on naïve mice (Fig. 6A). We then generated *ap-1*-knockdown ticks by microinjecting *ap-1*-dsRNA into naïve ticks. Microinjections were performed as described in methods and in our previous studies^12,15,16^. QRT-PCR assays revealed no significant difference (P > 0.05) in the level of *ap-1* transcripts between buffer-injected mock and control dsRNA (prepared from multiple cloning site of pL4440 vector)-injected mock controls (Supplementary Fig. 9B)-injected ticks. QRT-PCR assays showed approximately two-three fold reduced (P < 0.05) levels of *ap-1-* mRNA in the *ap-1*-knockdown ticks when compared to mock controls (Fig. 6B and Supplementary Fig. 9B). QRT-PCR assays also showed a three-four fold reduced (P < 0.05) levels of *iafgp* transcripts (Fig. 6C) and *A*. *phagocytophilum* burden (Fig. 6D) in *ap-1*-knockdown ticks when compared to mock controls. Furthermore, EMSAs performed with nuclear proteins isolated from *ap-1* knockdown fed nymphal ticks revealed approximately two to three fold decrease in *ap-1* probe shift in comparison to the shift observed with nuclear proteins isolated from mock control (Fig. 6E and Supplementary Fig. 10A,B). These results provide further evidence on the role of AP-1 as an upstream transcriptional activator in the regulation of *iafgp* gene expression during *A*. *phagocytophilum* acquisition by ticks.

### Knockdown of *ap-1* impacts *iafgp* expression and *A*. *phagocytophilum* burden in ISE6 tick cells

**Figure 7:**
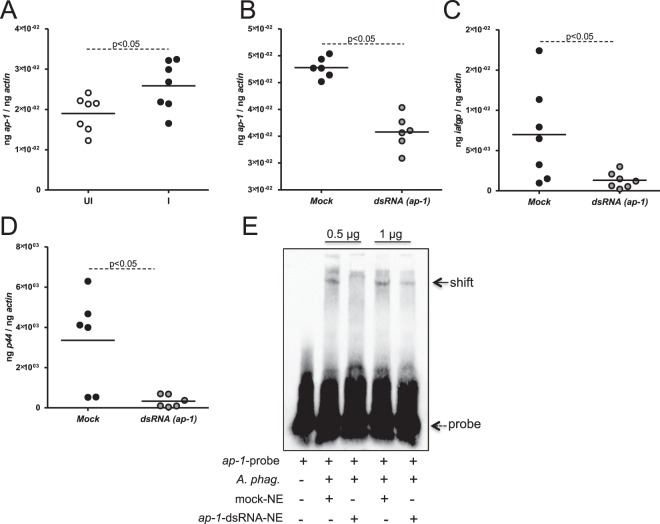
Knockdown of *ap-1* affects expression of *iafgp* gene and *A*. *phagocytophilum* burden in ISE6 cells *in vitro*. (**A**) QRT-PCR results showing *ap-1* transcripts in uninfected or *A*. *phagocytophilum*-infected ISE6 cells at 48 hrs p.i. The QRT-PCR results for the expression of *ap-1* (**B**) or *iafgp* (**C**) transcripts in tick cells upon treatment with mock or *ap-1*-dsRNA is shown. (**D**) Bacterial burden in mock or *ap*-1-silenced ISE6 cells at 24 hrs p.i. is shown. (**E**) Gel shift assays with biotinylated *iafgp*-promoter probe containing AP-1 binding site (DS704943, 18144–18193 bp) and nuclear proteins (0.5, 1 µg) prepared from *A*. *phagocytophilum*-infected mock or *ap-1*-silenced ISE6 cells is shown. Dotted arrow indicates free probe and solid arrow indicates shift. Each circle in panels A–D indicates RNA/DNA samples generated from one tick cells culture well. Statistical analysis was performed using Student’s t test and P value less than 0.05 was considered significant. NE indicates nuclear extracts and + or − indicates presence or absence, respectively.

We then analyzed *ap-1* transcripts in ISE6 *in vitro* tick cells. The levels of *ap-1* transcripts were significantly induced (P < 0.05) in *A*. *phagocytophilum-*infected ISE6 tick cells when compared to uninfected cells (Fig. 7A). RNAi-mediated silencing showed significantly reduced (P < 0.05) levels of *ap-1* transcripts in *ap-1* knockdown tick cells when compared to mock controls (Fig. 7B). Reduced (P < 0.05) *iafgp* mRNA levels was also evident in *ap-1* knockdown tick cells when compared to the levels noted in mock control (Fig. 7C). Furthermore, a dramatic reduction (P < 0.05) in *A*. *phagocytophilum* burden was observed in *ap-1* knockdown tick cells when compared to the bacterial burden in mock control (Fig. 7D). EMSAs performed with nuclear proteins isolated from *ap-1* knockdown tick cells revealed approximately two to three fold decrease in *ap-1* probe shift in comparison to the shift observed with nuclear proteins isolated from mock-treated tick cells (Fig. 7E and Supplementary Fig. 10C,D). Collectively, these results indicate an important role for AP-1 in *iafgp* gene regulation and survival of *A*. *phagocytophilum* in tick cells.

### AP-1 induces *iafgp* expression in unfed nymphs at cold temperatures

**Figure 8:**
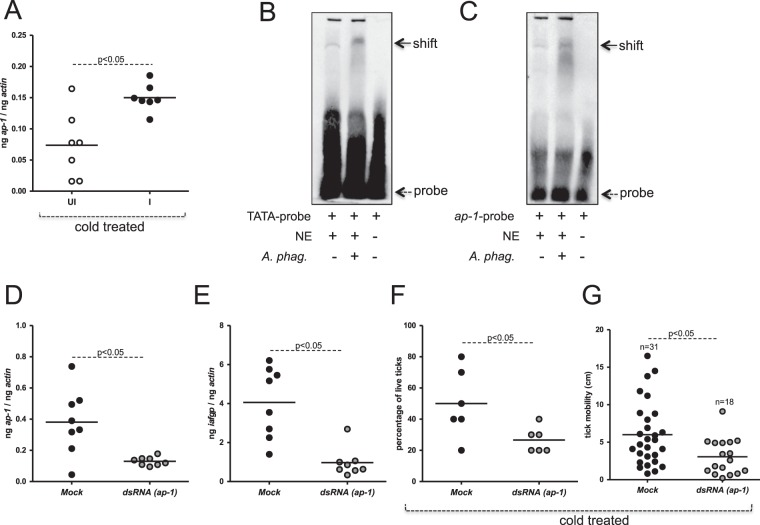
Silencing of *ap-1* in nymphs by RNAi reduces *iafgp* expression and survival of ticks at cold temperatures. (**A**) QRT-PCR results showing *ap-1* transcripts in uninfected or *A*. *phagocytophilum*-infected ticks incubated at 10 ± 1 °C for 8 hrs. Gel shift assays with biotinylated *iafgp* TATA (**B**) probe (DS704943, 18338–18387 bp) or probe containing AP-1 binding (**C**) site (DS704943, 18144–18193 bp) and nuclear proteins (2.5 µg) prepared from uninfected (UI) or *A*. *phagocytophilum*-infected (I) nymphs incubated at 10 ± 1 °C for 8 hrs is shown. Dotted arrow indicates free probe and solid arrow indicates shift. NE indicates nuclear extracts and + or − indicates presence or absence, respectively. QRT-PCR analysis showing reduced *ap-1* (**D**) or *iafgp* (**E**) mRNA levels in *ap-1*-dsRNA–injected uninfected nymphal ticks compared with the mock-treated controls. (**F**) Percentage survival of mock- or *ap-1*-dsRNA–injected nymphal ticks at the LT_50_ time point is shown. (**G**) Mobility (in cm) by mock- or *ap-1*-dsRNA–injected ticks at LT_50_ time point (−20 °C, 25 min) is shown. In panel A,D,E and G each circle represents one individual tick. Whereas, in panels F each circle represents one experiment performed with 10 ticks/group. Statistical analysis was performed using Student’s t test and P value less than 0.05 was considered significant in panels A and D–G.

We then tested whether *A*. *phagocytophilum*-infected ticks express higher level of *ap-1* transcripts at cold temperatures. A significant enhanced (P < 0.05) level of *ap-1* transcripts was noted in *A*. *phagocytophilum*-infected ticks when compared to uninfected controls upon incubation of these ticks at cold (10 ± 1 °C) temperature (Fig. 8A). In addition, *ap-1* transcripts were significantly upregulated upon incubation of ticks at cold (4 ± 1 °C) temperature in comparison to the levels noted in ticks incubated at 23 ± 1 °C (Supplementary Fig. 11A). EMSAs performed with total nuclear proteins isolated from *A*. *phagocytophilum*-infected ticks (incubated at cold temperature) revealed increased shift in *iafgp* TATA- (Fig. 8B and Supplementary Fig. 11B,C) and *ap-1* probes (Fig. 8C and Supplementary Fig. 11D,E) in comparison to the shifts observed upon incubation with extracts prepared from uninfected controls. These results further support *A*. *phagocytophilum* induced AP-1-mediated regulation of *iafgp* gene expression at cold temperatures.

### RNAi of *ap-1* affects tick survival in the cold

In our previous study, we reported that knockdown of *iafgp* expression affects survival of nymphs at cold temperature^12^. As *ap-1* is critical for *iafgp* expression, we now tested whether knockdown of *ap-1* impacts tick cold tolerance ability. Cold tolerance assays were performed as described in methods and in our previous study^12^. RNAi-mediated gene silencing showed a two-three fold reduced (P < 0.05) levels of *ap-1* mRNA in *ap-1* knockdown ticks in comparison to mock controls (Fig. 8D). In addition, three-four fold reduced (P < 0.05) level of *iafgp* mRNA was noted in *ap-1* knockdown ticks when compared to the levels noted in mock control (Fig. 8E). Similar observation was noted in *A*. *phagocytophilum*-infected *ap*-1 knockdown ticks (Supplementary Fig. 12A,B). The *ap-1* knockdown or mock control ticks were subjected to cold tolerance assays. Cold tolerance assays that revealed significant reduction in the survival of *ap-1* knockdown ticks when compared to mock control (Fig. 8F). A two-three fold reduced mobility was observed in *ap-1* knockdown ticks when compared to mock control upon treatment of ticks to cold temperatures (Fig. 8G). Similar observations were evident with *A*. *phagocytophilum*-infected *ap-1* knockdown ticks (Supplementary Fig. 12C,D). Collectively, these results suggest that arthropod transcriptional activator AP-1-mediated regulation of *iafgp* gene expression is critical for the survival of *A*. *phagocytophilum* and its vector in the cold.

## Discussion

**Figure 9:**
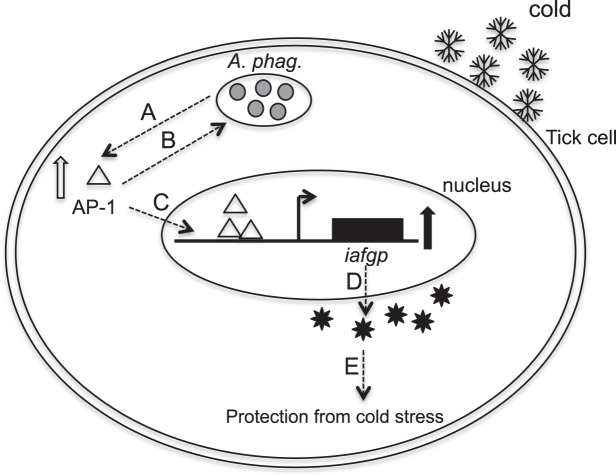
Model showing *A*. *phagocytophilum* mediated AP-1-IAFGP signaling in tick cold tolerance. (**A**) Upon infection, *A*. *phagocytophilum* (shown as morulae within a tick cell) and cold shock upregulate *ap-1* gene expression leading to increased production of AP-1 protein. (**B**) Increased production of AP-1 promotes *A*. *phagocytophilum* survival in ticks. In addition, AP-1 translocates into the nucleus and binds to *iafgp* promoter (**C**) to increase tick antifreeze gene expression leading to increased production of IAFGP (**D**) protein. (**E**) Increased production of IAFGP protects *A*. *phagocytophilum* and tick cells from cold. Schematic representation is not to the scale.

Several studies are focused on understanding molecular cellular signaling in blood sucking arthropods upon infection with various pathogens^21,40–46^. Our previous study showed an important role for tick antifreeze glycoprotein, IAFGP, in the survival of *A*. *phagocytophilum* and its vector host in the cold^12^. In this study, we provide evidence that *A*. *phagocytophilum* modulate arthropod fundamental transcription factor, AP-1, as an upstream activator for regulating tick *iafgp* gene expression critical for their survival in the cold (Fig. 9). Our study provides a novel model (Fig. 9) of molecular-cell signaling in mediating vector-pathogen interactions in the cold.

The domain analysis revealed presence of bZIP domain at the C-terminus region of *I*. *scapularis* AP-1. The bZIP domain-containing proteins are present in wide range of eukaryotic proteins (including AP-1 family proteins) that are recognized as DNA-binding transcription factors^47^. Ticks belonging to Ixodidae family are medically important vectors that transmit various pathogens to humans^1^. High degree of conservation across the entire *I*. *scapularis* AP-1, in particular at bZIP domain region, with AP-1 orthologs from other Ixodidae ticks suggests a possible conserved role for arthropod AP-1 in tick-pathogen interactions. Our results from domain analysis, ClustalW alignments and phylogenetic analysis provides an opportunity for future studies in characterizing various roles for arthropod AP-1 in tick-pathogen interactions across the Ixodidae family.

Upon entry into ticks, *A*. *phagocytophilum* colonizes salivary glands^48^. The upregulation of AP-1 in unfed nymphal tick salivary gland suggests a role for this molecule in *A*. *phagocytophilum* colonization in ticks. In addition, the results from RNAi analysis suggest that AP-1-mediated upregulation of *iafgp* gene expression is important in the acquisition of *A*. *phagocytophilum* by naïve ticks. This observation supports our previous finding that reported reduced *A*. *phagocytophilum* loads in *iafgp*-knockdown ticks upon feeding on an infected murine host^12^. Several experiments including EMSAs with recombinant AP-1 protein and with nuclear extracts generated from *ap-1*-dsRNA ticks directly support the role of AP-1 as a transcriptional activator in the regulation of *iafgp* promoter.

We considered region of ~700 bp as a putative promoter for *iafgp* gene, because it contained both the tested regions (TATA and AP-1 binding sites). The observation of no shift with the other tested AP-1 probe (region corresponding to 18032–18081 in GenBank acc. no. DS704943) and HSF-1 probe (region corresponding to 18093–18142 in GenBank acc. no. DS704943) upon incubation with nuclear proteins isolated from uninfected or *A*. *phagocytophilum*-infected nymphs suggests a non-definitive sites for AP-1 or HSF-1 binding, respectively and may not be important for bacterial-mediated influence on *iafgp* gene expression. The transfection of pGLuc construct containing P_*iafgp*_ with all tested sites (two AP-1 sites, one HSF-1 site and one TATA site) that showed increased promoter activity in comparison to the control clearly supports that the ~700 bp is the putative *iafgp* promoter. However, it is not surprising to hypothesize that ~320 bp region (18144–18463 bp in GenBank acc. no. DS704943) that contain one each of confirmed AP-1 and TATA site is sufficient for *iafgp* expression. Future studies would reveal whether ~320 bp promoter region is sufficient to direct *iafgp* gene expression.

Bioinformatics analysis also revealed presence of more than 100 transcription factors on *iafgp* promoter. These transcription factors include but not limited to HSF-2 sites. The HSF-1 probe used in this study also contained a putative HSF-2 binding site, thus, excluding the possibility of the later protein to bind *iafgp* promoter at that predicted site. Studies have revealed that genes containing TATA-box promoters are associated with stress and are highly regulated in comparison to the genes that are TATA-less promoters^37,49^. As *iafgp* is a stress-induced (infection and cold) gene with a strong TATA-box, it is reasonable to hypothesize that several transcription factors could bind on different regions of the *iafgp* promoter. However, the presence of strong binding site in the *iafgp* promoter (Fig. 3), a role in stress^35^ and in interactions with pathogens^30,34,35^ strongly supports that AP-1 is an important transcriptional activator of tick antifreeze gene expression. Our previous study reported OATP-KAT pathway in the survival of *A*. *phagocytophilum* in ticks^16^. The observation of AP-1 binding to the KAT promoter further suggests that *A*. *phagocytophilum* modulate AP-1 as a central player to control downstream tick genes critical for its survival in ticks. Our future studies would reveal interesting aspects on the role of any other possible *I*. *scapularis* transcription factors in the regulation of *iafgp* gene or in the interactions of AP-1 with the *iafgp* or other tick promoters.

The observation of reduced survival of *ap-1-*dsRNA ticks at cold temperatures suggests that lack of AP-1 impacts *iafgp* gene expression. In due consideration of AP-1 as a global transcriptional regulator, it is possible to reason that silencing of *ap-1* gene expression could impact other tick cold tolerance strategies. However, the observation of reduced *iafgp* gene expression at cold temperatures due to *ap-1* silencing that subsequently affected tick ability to survive cold strongly supports the role of AP-1-IAFGP signaling in the survival of ticks at cold temperatures. This notion is further supported by our previous study that reported reduced ability of *iafgp*-deficient ticks to survive at cold temperature^12^.

In summary, we show that *A*. *phagocytophilum* modulates arthropod AP-1 to activate tick antifreeze gene expression facilitating survival of both the rickettsial pathogen and its vector host in the cold. Understanding detailed molecular signaling, such as this, in vector-pathogen interactions would serve as an important means for developing new strategies to combat several rickettsial vector-borne diseases.

## Methods

### *A*. *phagocytophilum*, ticks and ISE6 cell line

The NCH-1 isolate of *A*. *phagocytophilum* was used in generating infected ticks as described^16^. The HZ isolate of *A*. *phagocytophilum* was used in RNAi and *in vitro* ISE6 cell line experiments. Both isolates are mentioned as *A*. *phagocytophilum*. The pGLuc constructs were transformed in to *Escherichia coli* DH5α strain for maintenance and amplification. *E*. *coli* BL21 strain was used for recombinant AP-1 protein purification. Uninfected *I*. *scapularis* ticks (larvae and nymphs) were obtained from Connecticut Agricultural Experiment Station (New Haven, CT). AP-1 knockdown experiments were performed in ticks obtained from BEI Resources, NIAID, NIH. The ISE6 cell line was obtained from Dr. Ulrike Munderloh and was propagated as reported^50^. The *A*. *phagocytophilum* isolate HZ was maintained in human promyelocytic cell line (HL-60, American Type Culture Collection, USA) as reported in our previous study^16^. Ticks were housed in the controlled environment chamber (Parameter Generation and Control, Black Mountain, NC) with settings for temperature at 23 ± 2 °C, humidity at 90–93% and photoperiod time set for 14 hrs light and 10 hrs dark exposure.

### Animals

All animal experiments in this study were performed with female C3H/HeN mice (4–6 weeks old). These mice were purchased from Charles River Laboratories, USA. Unfed nymphs were generated by feeding larvae on either uninfected or *A*. *phagocytophilum*–infected mice. The fed larvae were housed in environment chamber (with settings described in the earlier section) for molting to nymphs. In knockdown assays, *ap-1-*dsRNA was microinjected into uninfected nymphs and were allowed to feed on *A*. *phagocytophilum*-infected mice. Mock injections were performed in similar way in uninfected ticks with either a dsRNA fragment generated from multiple cloning site of pL4440 vector or buffer solution and were processed as controls. The engorged and repleted ticks were collected and RNA or DNA extractions were performed. QRT-PCR analysis was carried out on these tick samples to evaluate knockdown efficiency, bacterial burden and gene expression. The mice studies were performed based on animal protocol 16–017 approved by the Old Dominion University Institutional Animal Care and Use Committee (IACUC) as reported previously^16^. Animal husbandry and administration of tranquilizer during animal experiments was performed as reported previously^16^.

### Prediction of *iafgp* or *kat* promoter regions and phylogenetic analysis

The GenBank acc. no. DS704943 nucleotide sequence was downloaded from National Center for Biotechnology Information (NCBI) and region corresponding to −692 to +15 bp from the start of *iafgp* CDS or approximately 1000 bp region upstream of *kat* promoter was analyzed for the prediction of TATA box and putative transcription factors. The TATA box was predicted at HCtata, Neural network promoter prediction and TSSW servers as described in our previous publication^15^. Results from three predictions were compared and TATA box site mapped by all three software’s were considered for EMSA probe design. Putative transcription factor binding sites were predicted using online software TFBIND^36^ and obtained results were compared with the consensus sequences of transcription factor binding sites. The high match between TFBIND prediction and known consensus sequences was considered for the EMSA probe design and prediction of putative transcription factor binding sites on the *iafgp* promoter.

The phylogenetic analysis for AP-1 sequences is a rooted tree generated based on CLUSTALW alignment of amino acid sequences in DNASTAR software. Based on the software instructions, Kimura distance formula was used to calculate the distance values. In the phylogenetic analysis, the length of branches indicates distance between sequence pairs and units indicates number of substitution events. Scale at the bottom denotes amino acid substitutions per 100 amino acid residues.

### Quantification of gene expression and bacterial burden

Quantitative real-time PCR (QRT-PCR) analysis was carried out as reported^51–53^. Briefly, total RNA from different developmental stages of ticks (unfed larvae, nymphs, adults ticks), fed uninfected or *A*. *phagocytophilum*-infected nymphs and from ISE6 cells was extracted and processed for cDNA synthesis and QRT-PCR as reported previously^16,51,52^. Supplementary Table 1 contains sequences for the oligonucleotides used in this study. The *ap-1 or iafgp* mRNA levels or bacterial burden in samples were normalized against tick *beta-actin* mRNA or DNA level. *A*. *phagocytophilum* burden was quantified from genomic DNA isolated from infected unfed/fed nymphal ticks or tick cells. Total genomic DNA extractions were performed as described^16^ and processed for detection of *A*. *phagocytophilum p44* with primers mentioned in Supplementary Table 1. A standard curve from six 10-fold dilutions (1–0.00001 ng) of *ap-1*, *iafgp*, *p44* or actin was generated and used in the QRT-PCR analysis.

### RNAi experiments in ticks

The *ap-1-*dsRNA was generated as reported previously^12,15,16^. PCRs were performed (for the amplification of *ap-1* fragment) with oligonucleotides (Supplementary Table 1) containing two restriction enzyme sites (BglII and KpnI). The PCR fragment containing *ap-1* gene sequence was later gel purified using gel extraction kit (Qiagen, USA) and digested with BglII-KpnI restriction enzymes. Ligations were performed at BglII-KpnI digested pL4440 vector as reported previously^12,15,16^. Transformations were performed in *E*. *coli* DH5α cells and upon confirmation of the clone the construct was used for synthesizing dsRNA using MEGAscript RNAi Kit (Ambion Inc. USA). Microinjections with *ap-1* dsRNA, or dsRNA fragment generated from multiple cloning site of pL4440 vector or buffer solution were performed as described^12,15,16^. After microinjection, ticks were incubated for 24 hrs in a desiccator (for recovery) that was housed in an walk in-incubator with settings of temperature at 23 ± 2 °C, humidity at 90–93% and photoperiod time set at 14 hrs light and 10 hrs dark exposure. Microinjected ticks were later fed on uninfected or *A*. *phagocytophilum*-infected mice. Engorged ticks were collected after repletion and RNA or DNA extractions were performed. QRT-PCR was performed to determine silencing efficiency, gene expression and bacterial burden. For some experiments, unfed ticks injected with *ap-1-*dsRNA or mock were directly used for RNA or DNA extractions after 24 hrs incubation in the desiccator.

### RNAi experiments, transfection and luciferase assays in tick cell line

Silencing or transfection experiments in tick cells were reported previously^16^. 1 × 10^5^ tick cells were seeded onto 12 well plates in L-15B300 medium. The plates were then incubated for 24 hrs. After incubation, 750 ng of *ap-1* dsRNA or equal volume of mock solution or control dsRNA or pGLuc/pGLuc-P_*iafgp*_ constructs were mixed with Lipofectamine 2000 (Thermo Fisher Scientific, USA) reagent and added to the wells. The plates were then incubated for 6 hrs. A 2X L15-B300 medium was added after 6 hrs incubation and plates were then incubated for additional 18 hrs. *A*. *phagocytophilum* HZ strain was used in all these experiments. After 24 hrs, cell-free bacteria isolated from *A*. *phagocytophilum*-infected HL-60 cells were added. After 24 hrs post infection (p.i.), tick cells were collected and RNA or DNA extractions were performed. pGLuc-P_*iafgp*_ construct was prepared by cloning ~700 bp fragment (region corresponding to 17757–18463 in GenBank acc. no. DS704943) into the pGLuc vector. Briefly, ~700 bp fragment was PCR amplified using oligonucleotides containing EcoRI and HindIII restriction sites. The PCR fragment was digested and ligated into EcoRI and HindIII digested pGLuc vector. The ligated mix was transformed into *E*. *coli* DH5α strain and clones were selected on Luria-Bertani (LB) agar plates containing Ampicillin (50 μg/ml). Plasmids from clones were isolated using Qiagen Plasmid maxi kit (Qiagen, USA), sequenced and then used for transfection. Luciferase assays were performed with culture supernatant collected after 24 hrs post infection and 48 hrs post transfection using BioLux Gaussia Luciferase assay kit (New England Laboratories, USA) following manufacturer’s recommendations. Luminescence was measured using TECAN Plate reader (Tecan, USA).

### Electrophoretic mobility shift (EMSA) or gel shift assay

EMSAs were performed as described^15,16^. Nuclear proteins from unfed or fed uninfected or *A*. *phagocytophilum*-infected ticks or tick cells were extracted as described^15,16^. For cold treatment experiments, nymphal unfed ticks were incubated at 10 ± 1 °C for 8 hrs and then processed for the preparation of nuclear extracts. The total protein concentration in the nuclear extracts was measured by BCA kit (Pierce, USA) and mentioned in the figure legends. Sequences for the probes were designed based on the bioinformatics prediction using tools as described^15^. Probes for EMSAs were prepared by annealing complimentary oligonucleotides containing *iafgp* TATA region or AP-1 or HSF-1 binding sites. Biotin labeling of oligonucleotides was performed as described^15,16^. EMSAs were performed on 6% polyacrylamide gels as reported previously^15,16^. After run, samples from gels were transferred and processed for detection of gel shifts using chemiluminescent detection method as reported previously^15,16^. Densitometry analysis was performed as described^52^. ChemiDoc MP imager (BioRad, USA) was used for obtaining gel images.

### Tick cold tolerance and mobility assays

Tick cold tolerance assays and mobility determination was performed as described^12^. Briefly, *ap-1-*dsRNA or mock-treated unfed uninfected or *A*. *phagocytophilum*-infected nymphs were incubated at 23 ± 1 °C for 24 hrs in a desiccator and were later split into 10 ticks/group in plastic vials with ventilated covers to allow aeration. These vials were then immediately placed at −20 °C on a flat surface for 25 minutes (min). Vials were immediately shifted to 4 °C for 1 h and then later incubated at room temperature for additional 1 h to reacclimatize ticks. The reacclimatized ticks were given a breath test and then immediately placed in a side-by-side (1 cm apart) in a line on Whatman filter paper as described in our previous study^12^. Any tick moving out of the line within 10 min was considered to have survived the cold temperature. A total of six independent experiments (with 10 ticks/group/experiment) for uninfected ticks and three independent experiments (10 ticks/group/experiment) for *A*. *phagocytophilum* infected ticks were performed. Percentage of tick survival at −20 °C was determined for each experiment. Mobility of ticks after rapid cold shock was determined by measuring distance traveled by each tick on the Whatman paper. Distance from the start point to the end point was measured in centimeters for each individual tick.

### AP-1 protein expression and purification

Full length AP-1 protein purification was performed as described^54^. Full-length *ap-1* sequence was amplified using oligonucleotides containing BamHI and NotI sites. The oligonucleotide sequences are mentioned in Supplementary Table S1. Full-length PCR product was digested with BamHI-NotI restriction enzymes and ligated in-frame at BamHI-NotI digested pGEX vector (Amerhsam, USA). Empty vector without *ap-1* sequence was used as control. Ligation mix was transformed into *E*. *coli* BL21 chemical competent cells and clones were selected on LB agar plates containing ampicillin antibiotic (50 μg/ml). The rAP-1-GST or rGST alone were purified from BL21 cells following Hook GST protein Spin purification kit-Bacteria (G-Biosciences Inc. USA) as described^54^. Concentrations were measured using Pierce BCA protein measurement kit (ThermoFisher Scientific, USA).

### Tick tissue isolation

Tick tissues such as salivary glands and guts from each tick were isolated as reported^15,16^. The tissues were homogenized in lysis buffer and processed for RNA extractions. The cDNA was used in the QRT-PCR assays.

### Nucleotide or amino acid accession numbers

The following are the GenBank accession numbers used in this study: DS704943 (*iafgp* genomic region), XP_002404571 (*I*. *scapularis* AP-1), XM_002404527 (*I*. *scapularis ap-1* mRNA), JAP70916 (*I*. *ricinus* AP-1), JAC33128 (*A*. *triste* AP-1), AIT40211 (*R*. *microplus* c-Jun AP-1), NP_068607 (*R*. *norvegicus* AP-1), NP_034721 (*M*. *musculus* AP-1), NP_001252779 (*M*. *mulatta* AP-1) and NP_002219 (*H*. *sapiens* AP-1).

### Statistics

Statistical analysis was performed using GraphPad Prism6 software and Microsoft Excel 2016. The average means from the data analysis from two different samples was compared by non-paired Student t-test. The P value less than 0.05 was considered as significant throughout this study. Horizontal lines in the scatter plot represents mean of the readings. P values are indicated at the relevant places in the figures.

## Electronic supplementary material

Supplementary information
