# Supplementary material for: Arthropod transcriptional activator protein-1 (AP-1) aids tick-rickettsial pathogen survival in the cold
Source: Sci Rep. 2018 Jul 30;8:11409. doi: 10.1038/s41598-018-29654-6 (PMC6065373; doi:10.1038/s41598-018-29654-6)
Supplement: Supplementary file 1 — Supplementary information [file 41598_2018_29654_MOESM1_ESM.pdf]

Arthropod transcriptional activator protein-1 (AP-1) aids tick-rickettsial pathogen survival in the cold

Supreet Khanal [1], Vikas Taank [1], John F. Anderson [2], Hameeda Sultana [1, 3], and Girish Neelakanta [1, 3, #]

Supplementary information

Supplementary Figure legends

Supplementary Figure 1. Activator protein *ap-1* mRNA is upregulated in *A. phagocytophilum*-infected nymphal guts. QRT-PCR assays showing levels of *ap-1* transcripts in uninfected (UI) and *A. phagocytophilum*-infected (I) guts isolated from unfed nymphs is shown. Each circle represents one tick sample. The levels of *ap-1* transcripts were normalized to the levels of tick beta-actin transcripts. Statistical analysis was performed using Student's t test and P value less than 0.05 was considered significant.

Supplementary Figure 2. *A. phagocytophilum* and AP-1 influence *iafgp* promoter. A) EMSA gel image (different intensity of the image shown in Fig. 3A) showing increased shift of *iafgp* TATA-binding region promoter probe in the presence of *A. phagocytophilum*. EMSAs were performed with the biotin-labeled *iafgp* TATA-binding region promoter probe (DS704943, 18338-18387 bp) and uninfected or *A. phagocytophilum*–infected tick nuclear extracts. Wedges indicate increasing amounts of nuclear extracts (1, 3, 5 μg). B) Densitometry analysis for the gel

image in Fig. 3A is shown. C) EMSAs (different intensity of the gel image shown in Fig. 3B) performed with the biotin-labeled *iafgp* promoter probe containing AP-1 binding site (DS704943, 18144-18193 bp) and nuclear extracts (1, 2, 3 µg; wedges indicates increasing amounts of nuclear extract) prepared from uninfected or *A. phagocytophilum*–infected ticks. Dotted arrow indicates free probe and solid arrow indicates shift. NE indicates nuclear extracts. D) Densitometry analysis for the gel image in Fig. 3B is shown. Relative intensities of gel shifts were calculated to the control probe intensity in each gel image.

Supplementary Figure 3. *A. phagocytophilum* does not influence *iafgp* promoter via heat shock factor 1 (HSF-1). A) EMSA gel image showing no shifts in the *iafgp* promoter probes containing AP-1 binding site (DS704943, 18032-18081) in the presence of *A. phagocytophilum*. EMSAs were performed with the biotin-labeled probes and nuclear extracts prepared from uninfected or *A. phagocytophilum*–infected ticks. Wedges indicate increasing amounts of nuclear extracts (1, 3, 5 µg). Dotted arrow indicates free probe. NE indicates nuclear extracts. B) A different intensity for the EMSA gel image in A is shown. C) EMSA gel image showing no shifts in the *iafgp* promoter probes containing HSF-1 binding site (DS704943, 18093-18142) in the presence of *A. phagocytophilum*. EMSAs were performed with the biotin-labeled probes and nuclear extracts prepared from uninfected or *A. phagocytophilum*–infected ticks. Wedges indicate increasing amounts of nuclear extracts (1, 3, 5 µg). Dotted arrow indicates free probe. NE indicates nuclear extracts and + or – indicates presence or absence, respectively. D) A different intensity for the EMSA gel image in C is shown.

Supplementary Figure 4. Coomassie stained gel image showing the induction of rGST-AP-1 or rGST proteins in induced (ind) *E. coli* BL21 cell lysates. A) Uninduced (unind) or induced (ind) rGST-AP-1 or rGST *E. coli* BL21 cell lysates is shown. Purified recombinant proteins from *E. coli* BL21 cell lysates are also shown and indicated with solid arrow (rGST-AP-1) or dotted arrow (rGST). Appearance of a band at the size similar to GST in rGST-AP-1 lane indicates possible degradation of later protein. M indicates protein marker. B) A different intensity for the SDS-PAGE gel image in A is shown.

Supplementary Figure 5. *A. phagocytophilum* and rGST-AP-1 influence *iafgp* promoter. A) EMSAs (different intensity of the image shown in Fig. 3C) performed with the biotin-labeled *iafgp* promoter probe containing AP-1 binding site (DS704943, 18144-18193 bp) and rGST alone or rGST-AP-1 protein (1, 1.5 µg, wedges indicates increasing amounts of nuclear extracts). B) Densitometry analysis for the gel image in Fig. 3C is shown. C) EMSAs (different intensity of the image shown in Fig. 3D) performed with the biotin-labeled *iafgp* promoter probe containing AP-1 binding site (DS704943, 18144-18193 bp), recombinant GST or rGST-AP-1 protein (1.5 µg) and nuclear extracts (3 µg) prepared from uninfected (UI) or *A. phagocytophilum*–infected (I) ticks. Dotted arrow indicates free probe and solid arrow indicates shift. NE indicates nuclear extracts. D) Densitometry analysis for the gel image in Fig. 3D is shown. Relative intensities of gel shifts were calculated to the control probe intensity in each gel image.

Supplementary Figure 6. *A. phagocytophilum* and rGST-AP-1 influence *kat gene* promoter. EMSA (different intensity of the image shown in Fig. 4A) is shown. EMSAs performed with the biotinylated AP-1 region (DS929842, 124879-124830 bp) probe from *kat* putative promoter

containing AP-1 binding site (DS929842, 124858-124852 bp) and rGST alone or rGST-AP-1 protein (1, 3, 5 µg; wedges indicates increasing amounts of nuclear extracts). Dotted arrow indicates free probe and solid arrow indicates band shift.

Supplementary Figure 7. PCR amplification and sequencing of approximately 700 bp region of *iafgp* promoter. A) Agarose gel image showing PCR amplification of ~ 700 bp DNA fragment containing *iafgp* promoter from *I. scapularis* genomic DNA. The band was excised and cloned into promoterless pGLuc vector and sequenced. Oligonucleotides used in the PCR amplification are mentioned in Supplementary Table 1. Solid arrow indicates amplification product, dotted arrow indicates primer dimer, M indicates DNA marker and NTC indicates no-template control. B) Nucleotide sequence of the ~ 700 bp DNA fragment cloned in pGLuc vector is shown. The sequence is shown from 5'-3' direction. Length of the sequence is shown on one side of the sequence.

Supplementary Figure 8. pGLuc-P$_{iafgp}$ or pGLuc transfection showed no morphological changes in tick cells. Representative images (1 image for each group) of uninfected or *A. phagocytophilum*-infected pGLuc-P$_{iafgp}$ or pGLuc transfected tick cells after 48 h post transfection and 24 h post infection is shown. Scale in all images indicates 200 µm.

Supplementary Figure 9. Amplification of luciferase transcripts in tick cells. Agarose gel image showing luciferase amplicon from uninfected (UI) and *A. phagocytophilum*-infected (I) tick cells transfected with pGLuc-P$_{iafgp}$ or pGLuc is shown. Solid arrow indicates amplification product, dotted arrow indicates primer dimer, M indicates DNA marker and NTC indicates no-

template control. B) QRT-PCR analysis showing levels of *ap-1* transcripts in mock buffer injected (mock-EB) or mock-dsRNA (generated from multiple cloning site of pL4440 vector)-injected or *ap-1*-dsRNA-injected *A. phagocytophilum*-infected nymphs. Each circle represents one tick. In panel B, the level of *ap-1* transcripts was normalized to tick-beta actin levels. Statistical analysis was performed using Student's t test and P value less than 0.05 was considered significant in panel B.

Supplementary Figure 10. RNAi-mediated silencing of *ap-1* affects *A. phagocytophilum* mediated regulation of *iafgp* gene expression in ticks and tick cells. A) EMSAs (different intensity of the image shown in Fig. 6E) performed with the biotin-labeled *iafgp* promoter probe containing AP-1 binding site (DS704943, 18144-18193 bp) and nuclear extracts (2, 4, 6 µg: wedges indicates increasing concentration of nuclear extracts) prepared from *A. phagocytophilum*-infected mock or *ap-1*-dsRNA-treated ticks is shown. B) Densitometry analysis for the gel image in Fig. 6E is shown. C) EMSAs (different intensity of the image shown in Fig. 7E) performed with the biotin-labeled *iafgp* promoter probe containing AP-1 binding site (DS704943, 18144-18193 bp) and nuclear extracts (0.5, 1 µg; wedges indicates increasing concentration of nuclear extracts) prepared from *A. phagocytophilum*-infected mock or *ap-1*-dsRNA-treated tick cells is shown. Gel shifts and the free probes are indicated with arrows. NE indicates nuclear extracts. + indicates presence and – indicates absence. D) Densitometry analysis for the gel image in Fig. 7E is shown. Relative intensities of gel shifts were calculated to the control probe intensity in each gel image.

Supplementary Figure 11. *A. phagocytophilum* regulates *iafgp* expression in cold.

A) QRT-PCR analysis showing levels of *ap-1* transcripts in uninfected nymphs incubated at room temperature or at 4±1°C for 2 and 4 hours. The level of *ap-1* transcripts was normalized to tick-beta actin levels. Statistical analysis was performed using Student's t test and P value less than 0.05 was considered significant. B) EMSAs (different intensity of the images shown in Fig. 8B) performed with the biotin-labeled *iafgp* promoter TATA probe (DS704943, 18338-18387 bp) and nuclear extracts (2.5 µg) prepared from uninfected (UI) or *A. phagocytophilum*-infected (I) nymphal ticks incubated at 10±1°C for 8 hrs is shown. C) Densitometry analysis for the gel image in Fig. 8B is shown. D) EMSAs (different intensity of the images shown in Fig. 8C) performed with the biotin-labeled *iafgp* promoter probe containing AP-1-binding site (DS704943, 18144-18193 bp) and nuclear extracts (2.5 µg) prepared from uninfected (UI) or *A. phagocytophilum*-infected (I) nymphal ticks incubated at 10±1°C for 8 hrs is shown. E) Densitometry analysis for the gel image in Fig. 8C is shown. Relative intensities of gel shifts were calculated to the control probe intensity in each gel image. In panels B and D, dotted arrow indicates free probe and solid arrow indicates shift. NE indicates nuclear extracts and + indicates presence and – indicates absence.

Supplementary Figure 12. Silencing of *ap-1* by RNAi reduces *iafgp* expression and survival of *A. phagocytophilum*-infected ticks at cold temperature. QRT-PCR analysis showing reduced *ap-1* (A) or *iafgp* (B) mRNA levels in *ap-1*-dsRNA–injected *A. phagocytophilum*-infected unfed nymphal ticks compared with the mock-treated controls. C) Percentage survival of mock- or *ap-1*-dsRNA–injected *A. phagocytophilum*-infected ticks at the LT_{50} time point is shown. D) Tick mobility (in cm) by mock- or *ap-1*-dsRNA–injected *A. phagocytophilum*-infected ticks at LT_{50} time point (–20°C, 25 min) is shown. In panels A, B and D each circle

represents one individual tick. In, panel C each circle represents one experiment performed with 10 ticks/group. P value from non-paired Student's t-test is shown.

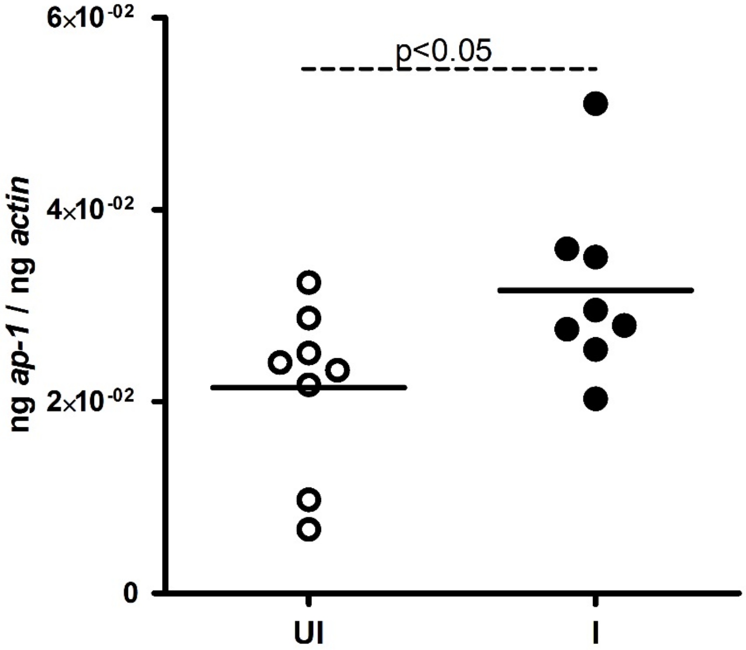

Supplementary Figure 1

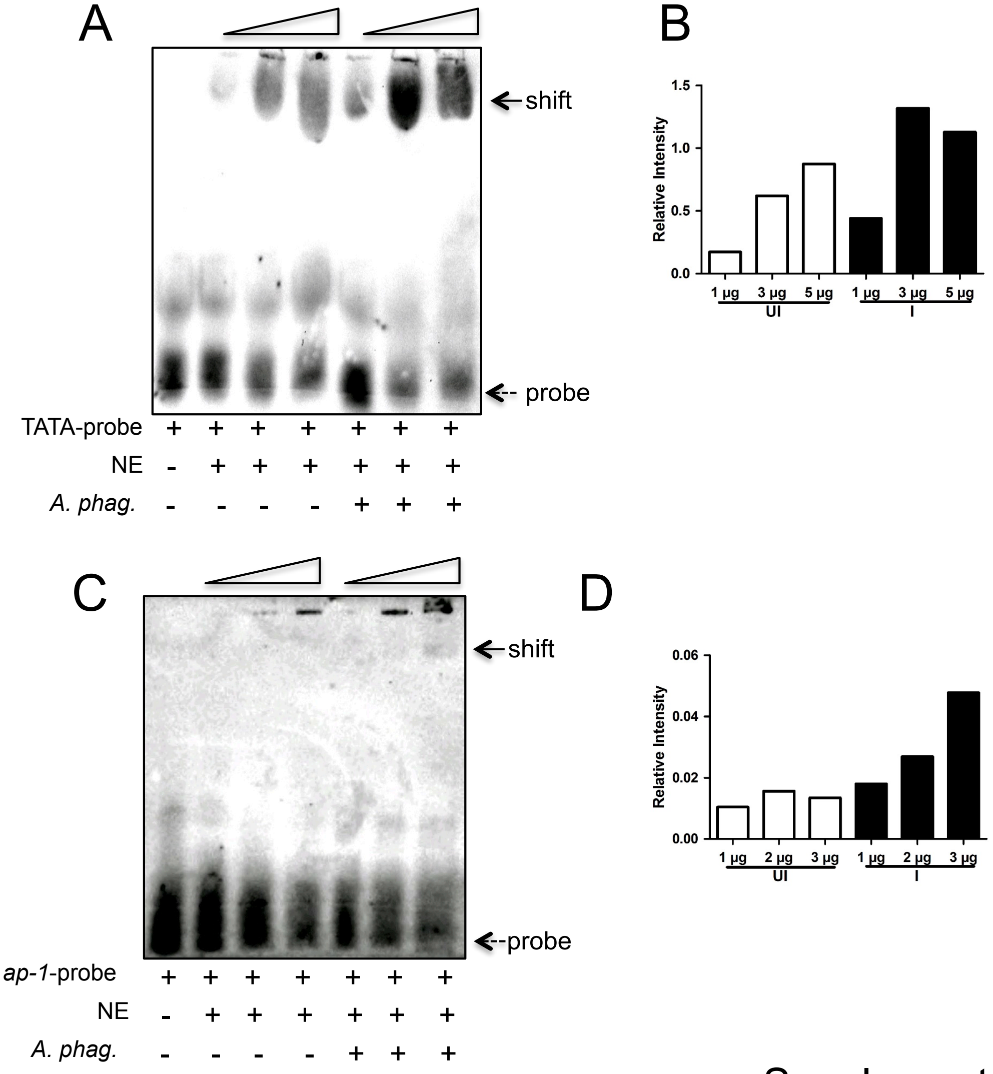

Supplementary Figure 2

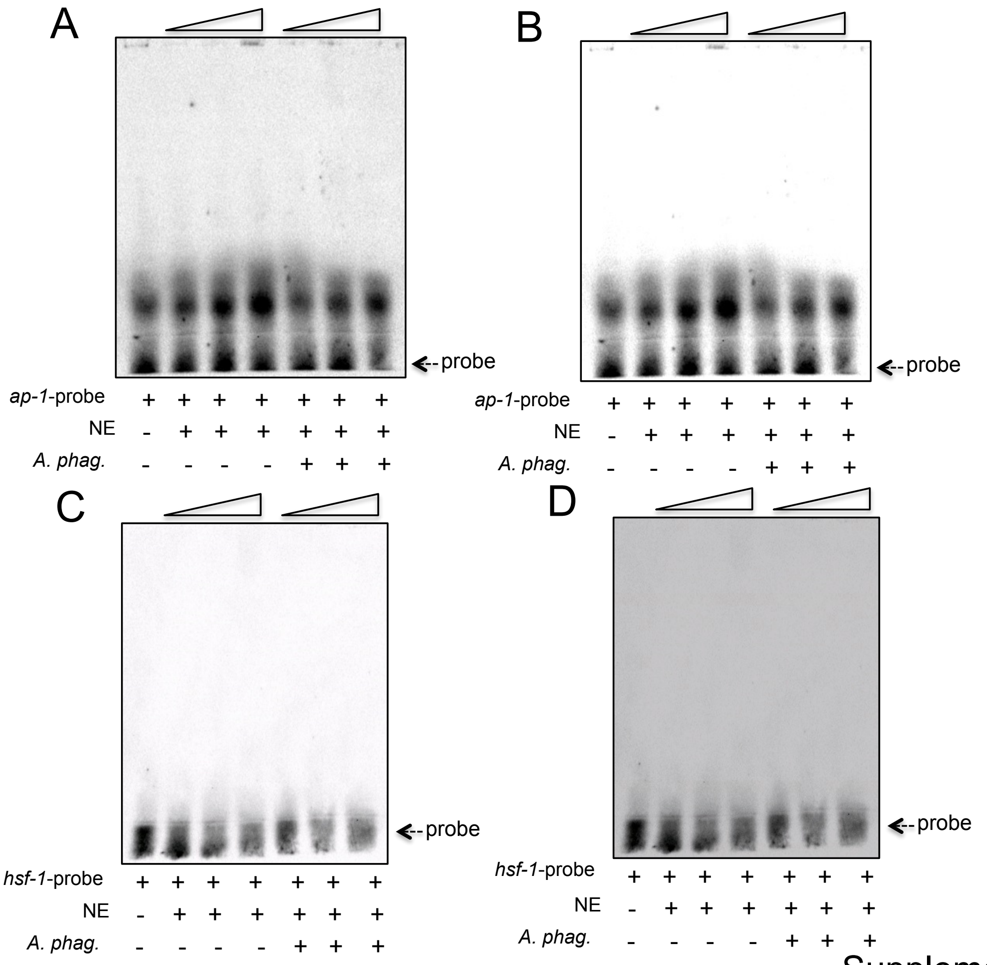

Supplementary Figure 3

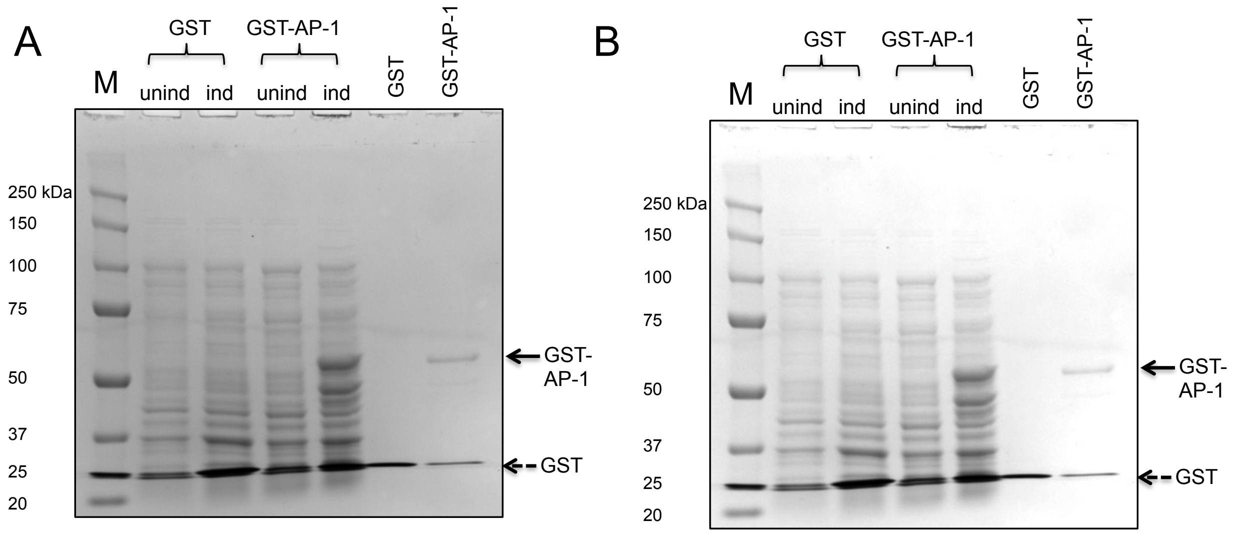

Supplementary Figure 4

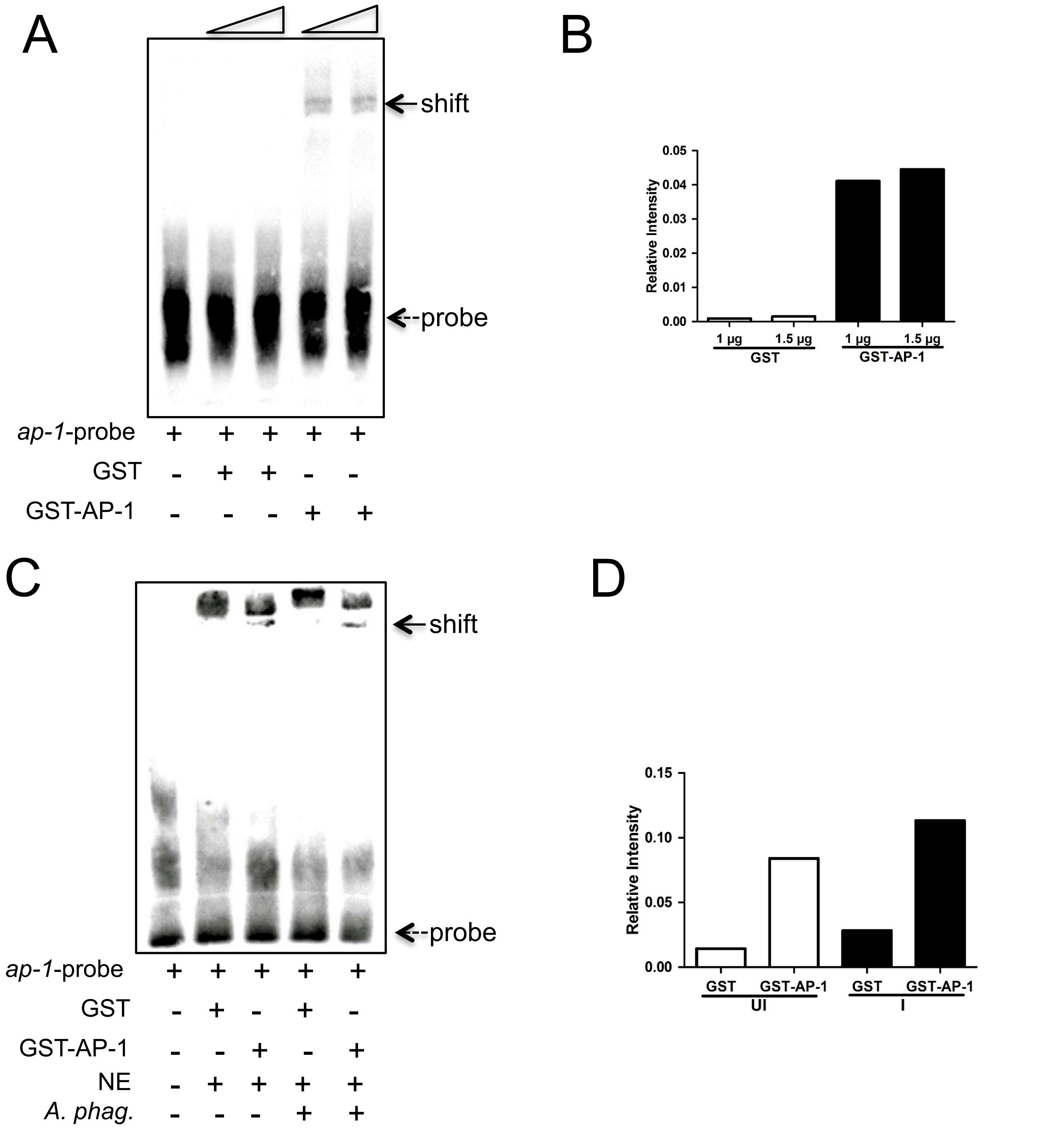

Supplementary Figure 5

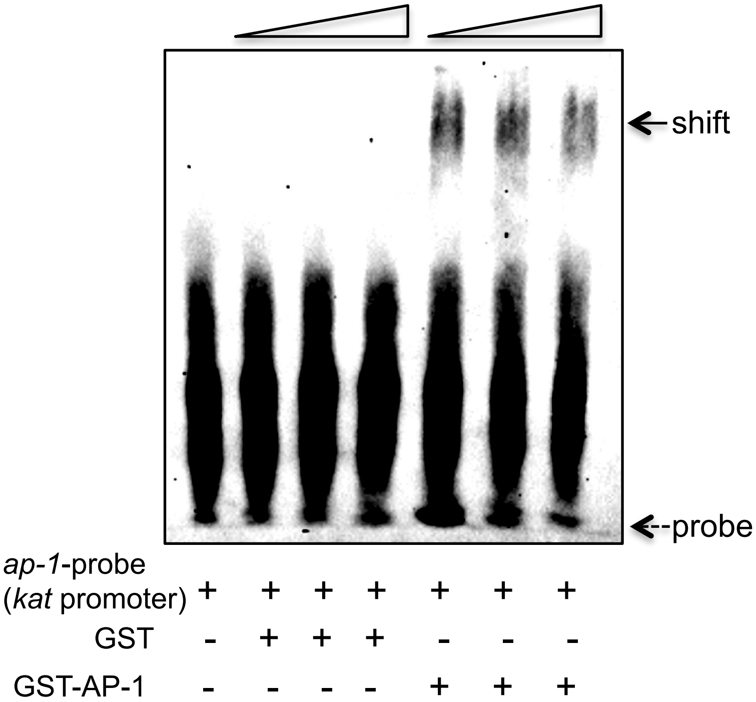

Supplementary Figure 6

A

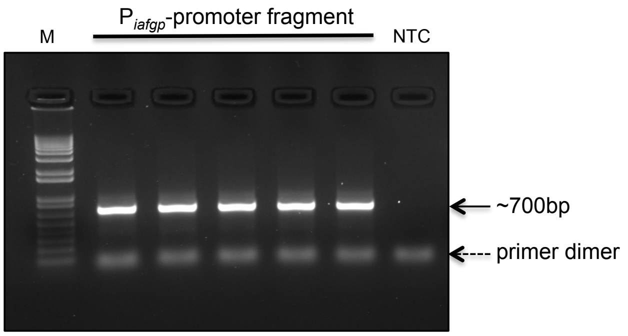

B

```
5'  CGCATACTCAGGTTAGAATAAAAAACAAGCCGTTTAAGCGGTGTTGTACACATTACTCCTCAAAAATAACGAGATACTCGTTACTTGACCTTAAAAGTAACTAAACACGT  110
5'  TACTTGTTACATAATATATTAACTGGGTTGAAGTAATTCGTTACTTGTAACGCGTTACGTAAATGTCTGGTCGAGGCAGTAGTTTCCGGTCGAGGTAGTTGACTTTGAGG  220
5'  CACGAGCCATCGCGCAGTGGGAGGACCGATGAGTCACCGGTATCGCGTGCATAGCACGTGGACACACATTCTGAGGTGACGCTCGGGAAGGCACTGAAGGGCAGGGAGCA  330
5'  GCACACGTGCTGCTCCCTGCCCTACTGTGCCTTCCGAGGAAAACACCTGCATAGCGAAAACGAGTGGATGAGTGGACTGTGTCGCAGCACAGCCGCCGAGCCAACGCCAC  440
5'  GTGTCGGCATTTTACTTACAACTACTACAGAATGCGATTAATACATGTCTGCATTTCAATTACAATCCGTGTCTTTTTTTGCAATTTTTATTGTAAAATTACATTCGCGT  550
5'  GGCAAAATATGAAGACAACTCGCCGTCCACCGCCAGGCTTATTTAGCTATATAAAACAACGCAAGCTCGCCGAAAGCACACGGGCACTACCAGACTTCAGCCAAGAGACT  660
5'  CCAAAGAAACCAACCTACTGAAAACCATGACGACTCTGGTG
```

Supplementary Figure 7

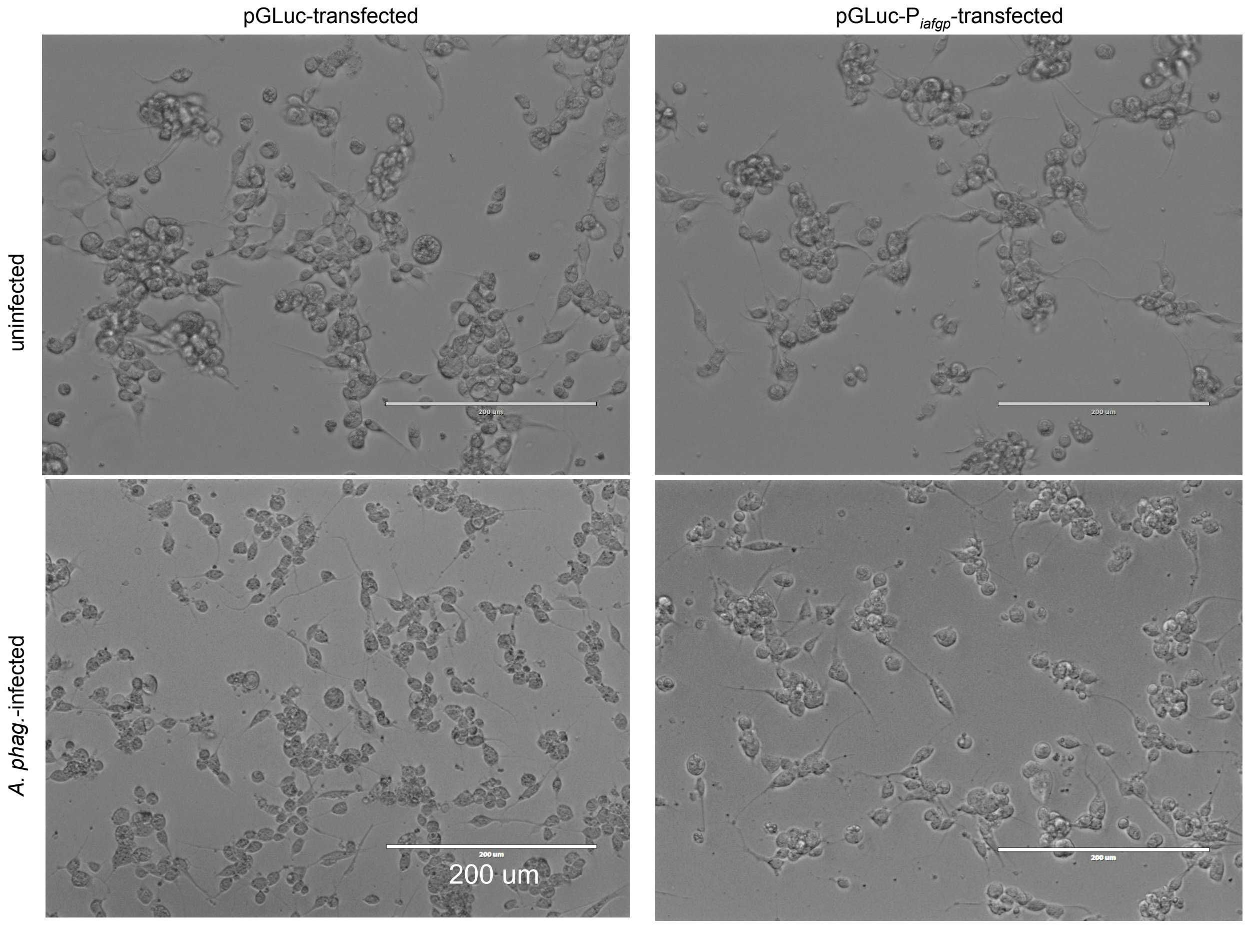

Supplementary Figure 8

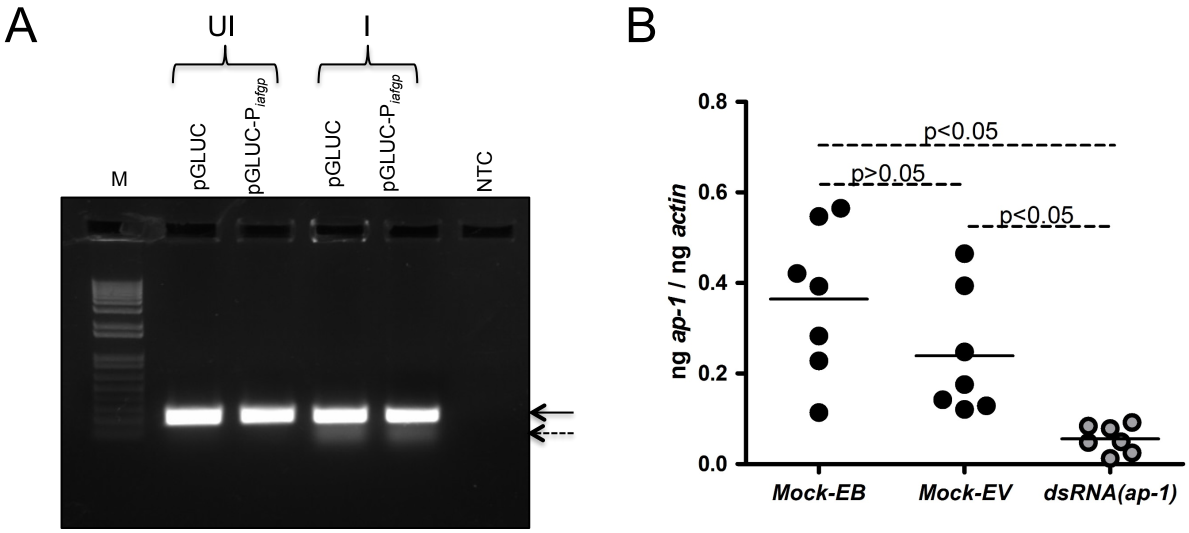

Supplementary Figure 9

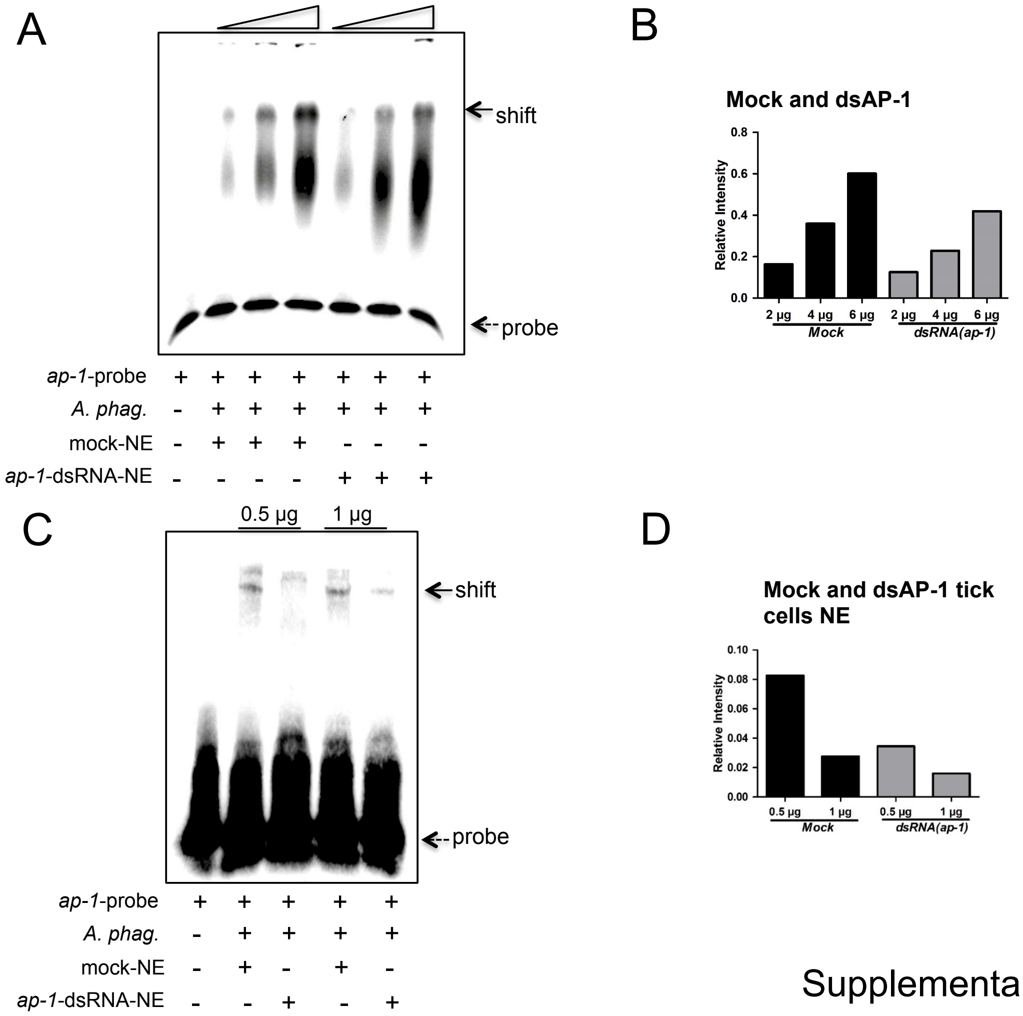

Supplementary Figure 10

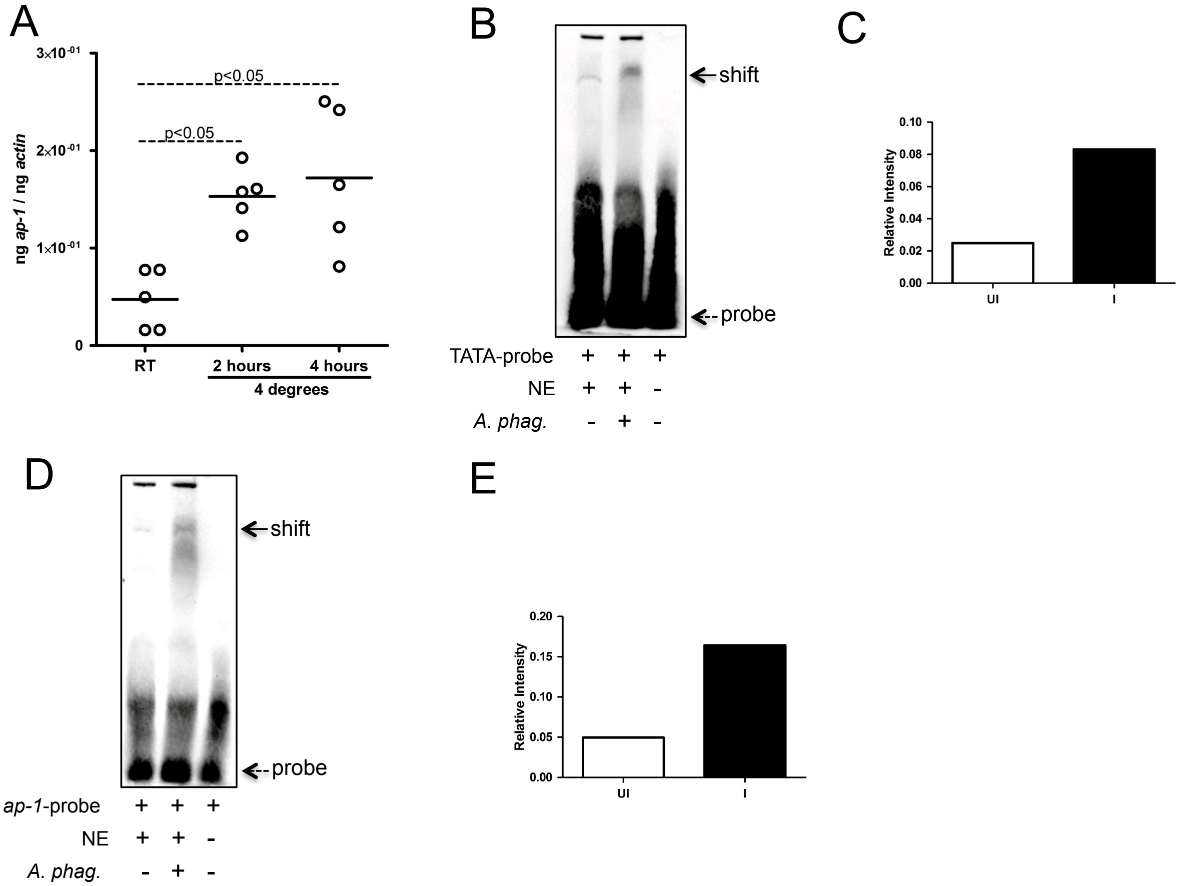

Supplementary Figure 11

213

214

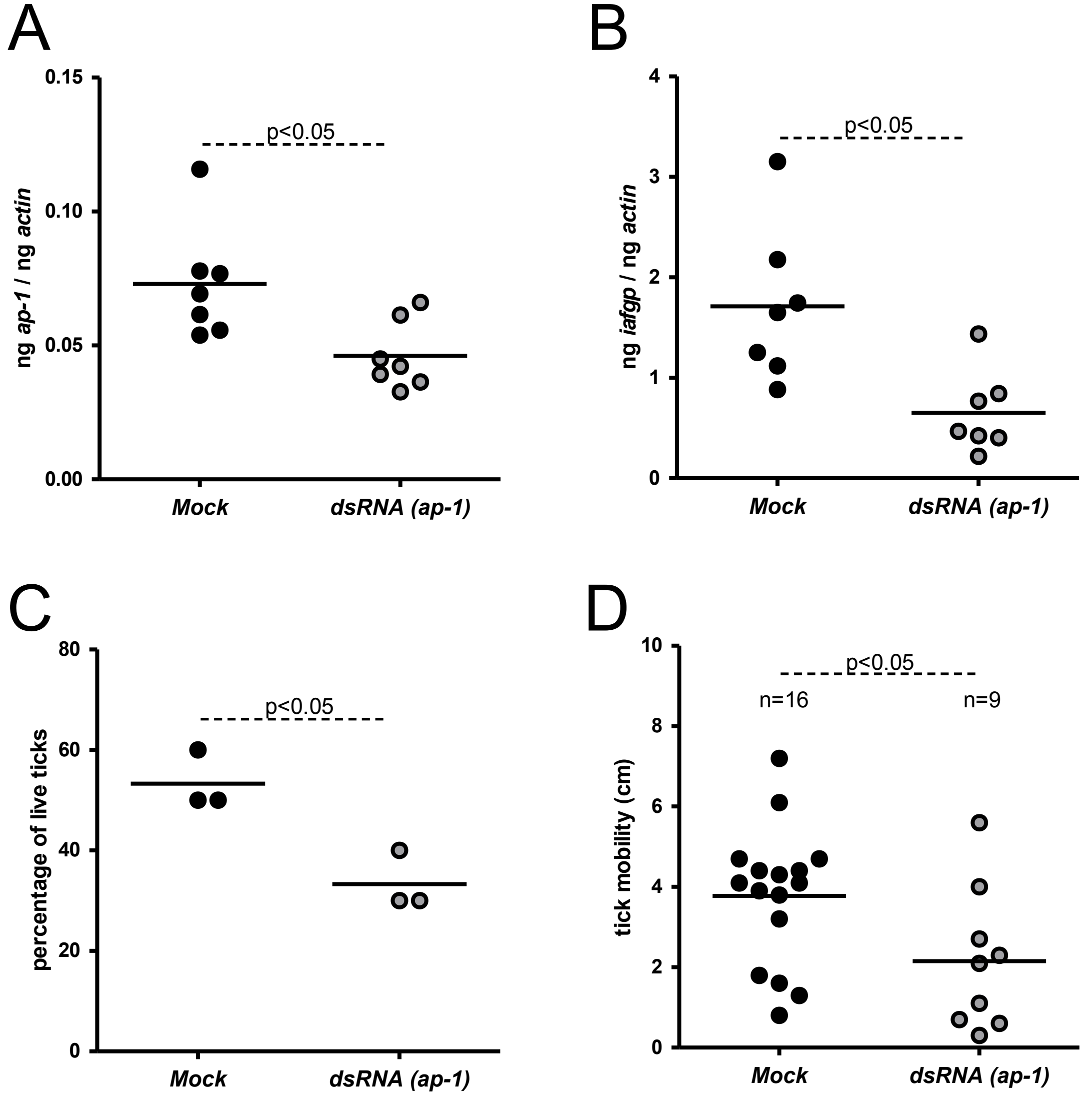

Supplementary Figure 12

Supplementary Table 1: Oligonucleotides used in this study

**Sequence (5'-3')**	**Purpose**
GGTATCGTGCTCGACTC	tick actin, QRT-PCR
CAGGGCGACGTAGCAG	tick actin, QRT-PCR
GTCGACGCGTTAGCCCAACT	*ap-1*, QRT-PCR
CGCCGCCGCCCAAAGA	*ap-1*, QRT-PCR
CTGCGCCATGGAGGAGTGTA	*iafgp*, QRT-PCR
CGTTATTTATTCCCACGTTTTCAT	*iafgp*, QRT-PCR
TGGGATCCATGACCCTGGACTTGAACAGTG	*ap-1,* pGEX-cloning
CGGCGGCCGCTCACGCCGTCGTCGCA	*ap-1*, pGEX-cloning
TGAGATCTCGTCGCCCGACCTGAACA	*ap-1*, RNAi
CGGGTACCCCGACGACGAGGGTAAGATGA	*ap-1*, RNAi
CCAGCGTTTAGCAAGATAAGAG	*Anaplasma*, QRT-PCR
GCCCAGTAACAACATCATAAGC	*Anaplasma*, QRT-PCR
TCCACCGCCAGGCTTATTTTGCTATATAATACAACGCA AGCTCGGCGAAA	*iafgp* TATA probe
TTTCGCCGAGCTTGCGTTGTATTATATAGCAAAATAA GCCTGGCGGTGGA	*iafgp* TATA probe
CACGTGGACACACATTCTGAGGTGACGCTCGGGAAAG CACAGAAGGGCAG	*iafgp ap-1* probe
CTGCCCTTCTGTGCTTTCCCGAGCGTCACCTCAGAATG TGTGTCCACGTG	*iafgp ap-1* probe
AAAACGAGTCGATGAGTGGACTGTGTCGCAGCACAGC CGCCGAGCCTACG	*iafgp ap-1* probe
CGTAGGCTCGGCGGCTGTGCTGCGACACAGTCCACTC ATCGACTCGTTTT	*iafgp ap-1* probe
CCCAAGCTTGCACCAGAGTCGTCATGGTTTTCAGT	P_{iafgp}, pGLUC cloning
CGGAATTCCGCATACTCAGGTTAGAATAAAAAACAAG	P_{iafgp}, pGLUC cloning
CGTGCTGCTCCCTGCCCTACTGTGCCTTCCGAGGAAA ACACCTGCATAGC	*iafgp*, HSF-1 probe
GCTATGCAGGTGTTTCCTCGGAAGGCACAGTAGGGC AGGGAGCAGCACG	*iafgp*, HSF-1 probe
GGGTTCCGCGCACATTTC	pGLUC sequencing
GATGCAGATCAGGGCAAACAGA	pGLUC sequencing
CCCAGGACGCTGCCACA	luciferase, QRT-PCR
CAGCCACTTCTTGAGCAGGTCA	luciferase, QRT-PCR
CGCAAGCATGATTGGACCAAGTGAATCAGGCGTAAG AAGTTGGTGATGGC	*kat* ap-1 probe
GCCATCACCAACTTCTTACGCCTGATTCACTTGGTCCA ATCATGCTTGCG	*kat* ap-1 probe
